# Single-Locus and Multi-Locus Genome-Wide Association Studies in the Genetic Dissection of Fiber Quality Traits in Upland Cotton (*Gossypium hirsutum* L.)

**DOI:** 10.3389/fpls.2018.01083

**Published:** 2018-08-17

**Authors:** Chengqi Li, Yuanzhi Fu, Runrun Sun, Yuanyuan Wang, Qinglian Wang

**Affiliations:** Collaborative Innovation Center of Modern Biological Breeding, School of Life Science and Technology, Henan Institute of Science and Technology, Xinxiang, China

**Keywords:** GWAS, multi-locus model, fiber quality, Upland cotton (*Gossypium hirsutum* L.), QTN, candidate gene

## Abstract

A major breeding target in Upland cotton (*Gossypium hirsutum* L.) is to improve the fiber quality. To address this issue, 169 diverse accessions, genotyped by 53,848 high-quality single-nucleotide polymorphisms (SNPs) and phenotyped in four environments, were used to conduct genome-wide association studies (GWASs) for fiber quality traits using three single-locus and three multi-locus models. As a result, 342 quantitative trait nucleotides (QTNs) controlling fiber quality traits were detected. Of the 342 QTNs, 84 were simultaneously detected in at least two environments or by at least two models, which include 29 for fiber length, 22 for fiber strength, 11 for fiber micronaire, 12 for fiber uniformity, and 10 for fiber elongation. Meanwhile, nine QTNs with 10% greater sizes (*R*^2^) were simultaneously detected in at least two environments and between single- and multi-locus models, which include TM80185 (D13) for fiber length, TM1386 (A1) and TM14462 (A6) for fiber strength, TM18616 (A7), TM54735 (D3), and TM79518 (D12) for fiber micronaire, TM77489 (D12) and TM81448 (D13) for fiber uniformity, and TM47772 (D1) for fiber elongation. This indicates the possibility of marker-assisted selection in future breeding programs. Among 455 genes within the linkage disequilibrium regions of the nine QTNs, 113 are potential candidate genes and four are promising candidate genes. These findings reveal the genetic control underlying fiber quality traits and provide insights into possible genetic improvements in Upland cotton fiber quality.

## Introduction

Cotton produces a fine natural fiber that is an important raw material for the textile industry. In recent years, technology development in the textile industry has been more rapid than improvements in the quality of cotton fiber, resulting in an inability to meet the industry needs, which include stronger, thinner, and more regular cotton fibers. China is the largest cotton producing country in the world, with the yield of Chinese cotton cultivars being equal to or slightly higher than those developed in the USA and Australia. However, the fiber qualities of the Chinese cotton cultivars, especially fiber strength (FS), are not as good (Wang et al., 2009). Upland cotton (*Gossypium hirsutum* L.) (2n = 4x = 52), one of the 50 *Gossypium* species and the leading natural fiber crop, produces more than 95% of the total cotton because of its high yield and wide adaptability (Chen et al., 2007). Improving the fiber quality is a major breeding target in Upland cotton.

Traditional breeding methods play important roles in cotton breeding. Predecessors bred a number of high-quality resource materials by hybridization, backcrossing, and other means using high fiber quality genes from Sea Island cotton (*Gossypium barbadense*) (Liang, 1999; Zhang et al., 2012). However, there still exists a negative correlation between fiber quality and yield, and complex correlated relationships among fiber quality traits (Miller and Rawlings, 1967; Smith and Coyle, 1997), which leads to the consequences that yield and quality, and individual fiber quality index, could not be simultaneously improved using traditional breeding strategies. The application of molecular markers that are closely linked to or significantly associated with the target quantitative trait loci (QTLs), for marker-assisted selection (MAS), can transform traditional phenotypic selection into direct genotypic selection, thereby improving the selection efficiency (Lee, 1995; Mohan et al., 1997). Therefore, it is important to elucidate the molecular genetics of cotton fiber qualities using molecular marker technology.

Association mapping based on linkage disequilibrium (LD) is a powerful tool for dissecting the genetic bases of complex plant traits. In contrast to the traditional linkage mapping, association mapping can effectively associate genotypes with phenotypes in natural populations and simultaneously detect many natural allelic variations in a single study (Huang and Han, 2014). Its high resolution, cost efficiency, and non-essential pedigrees have allowed association mapping to be applied in the dissection of many important cotton phenotypes, such as yield and its components (Mei et al., 2013; Zhang et al., 2013; Jia et al., 2014; Qin et al., 2015), fiber quality (Abdurakhmonov et al., 2008, 2009; Zhang et al., 2013; Cai et al., 2014; Qin et al., 2015; Nie et al., 2016), early maturity (Li et al., 2016a), disease resistance (Mei et al., 2014; Zhao et al., 2014), salt resistance (Saeed et al., 2014; Du et al., 2016), plant architecture (Li et al., 2016b), and seed quality (Liu et al., 2015). All of those studies, however, were based on using a limited number of simple sequence repeat markers (SSRs). The genetic bases of the quantitative traits could not be fully revealed at the genome-wide level.

As there is wide application of high-density genotyping platforms, the development of numerous single nucleotide polymorphism markers (SNPs) makes it possible to dissect the genetic architecture of quantitative traits through the genome-wide association studies (GWASs). Presently, GWAS has been successfully employed for several major crops, such as rice (Spindel et al., 2016), maize (Xu et al., 2017), wheat (Zegeye et al., 2014), barley (Visioni et al., 2013), oat (Newell et al., 2011), rapeseed (Zhou et al., 2017), soybean (Zhang J. et al., 2015), peanut (Zhang et al., 2017), and sorghum (Morris et al., 2013). For cotton fiber quality, Su et al. (2016b) performed a GWAS of fiber quality traits using 355 Upland cotton accessions and 81,675 SNPs developed from specific-locus amplified fragment sequences. They detected 16, 10, and 7 SNPs significantly associated with fiber length (FL), FS, and fiber uniformity (FU), respectively. In the study by Islam et al. (2016), the fiber quality data and 6,071 SNPs generated through genotyping-by-sequencing and 223 SSRs of 547 recombinant inbred lines were used to conduct a GWAS. One QTL cluster associated with four fiber quality traits, which include short fiber content, FS, FL, and FU, on chromosome A7 was identified and validated. Additionally, using the first commercial high-density CottonSNP63K array, Gapare et al. (2017) identified 17 and 50 significant SNP associations for FL and fiber micronaire (FM), respectively. Sun et al. (2017) and Huang et al. (2017) detected 46 and 79 significant SNPs, respectively, associated with several fiber quality traits. The above studies allowed the unraveling of the genetic architecture of fiber quality traits in cotton at the genome-wide level. However, the GWAS performed was based on the single-locus models, such as the general linear model (GLM) and the mixed linear model (MLM) (Bradbury et al., 2007). Multiple tests require that the test number undergoes a Bonferroni correction. The typical Bonferroni correction is often too conservative, which results in many important loci associated with the target traits being eliminated because they do not satisfy the stringent criterion of the significance test.

The multi-locus models are better alternatives for GWASs because they do not require the Bonferroni correction, and thus more marker-trait associations may be identified. Recently, several new multi-locus GWAS models, such as multi-locus RMLM (mrMLM, Wang et al., 2016), fast multi-locus random-SNP-effect EMMA (FASTmrEMMA, Wen et al., 2017), and Iterative modified-Sure Independence Screening EM-Bayesian LASSO (ISIS EM-BLASSO, Tamba et al., 2017), were developed. In this study, several models, including the single-locus and multi-locus models, were simultaneously used for the GWAS of fiber quality traits in Upland cotton based on a recently developed CottonSNP80K array (Cai et al., 2017), and the candidate genes were further identified. The results provide an insight into the complicated genetic architecture of the fiber quality traits in Upland cotton and reveal the whole-genome quantitative trait nucleotides (QTNs) for MAS in future breeding programs.

## Materials and methods

### Plant materials

A total of 169 Upland cotton accessions were examined in the present study, including 62 and 25 from ecological cotton-growing areas of the Yellow and Yangtze Rivers, respectively, in addition to 50 from Northwestern China, 22 from Northern China, and 10 from other countries (Supplementary Table S1). These accessions were elite cultivars originating in, or introduced to, China. All accessions showed stable inheritances after many generations of self-pollination.

### Experimental design and trait investigation

All materials were planted in the two different ecological cotton-growing areas of China, the Yellow River (Xinxiang City, Henan Province) and Northwestern China (Shihezi City, Xinjiang Province) during 2012 and 2013. The experiment adopted a randomized complete block design with single row plots and two replications. In Xinxiang, 14–16 plants were arranged in each row, with a row length of 5 m and a row interval of 1.0 m. In Shihezi, 38–40 plants were arranged in each row, with a row length of 5 m and a row interval of 0.45 m. Local normal management was carried out for all activities. For descriptive purposes, the four environments, 2012 Xinxiang, 2013 Xinxiang, 2012 Shihezi, and 2013 Shihezi, are designated as E1, E2, E3, and E4, respectively.

Lint fiber samples of ~15 g, taken from each row, were sent to the Fiber Quality Testing Center of the Institute of Cotton Research, Chinese Academy of Agricultural Sciences for the determination of fiber qualities (HVISPECTRUM, HVICC calibration level). Altogether, five fiber quality traits—FL (mm), FS (cN/Tex), FM, FU (%), and fiber elongation (FE, %), were investigated. To reduce environmental errors, the best linear unbiased predictors (BLUPs) for the five traits per genotype were estimated using the lme4 package (Bates et al., 2011). The BLUP values and single environments were used for the GWAS.

### SNP genotype calling

Genomic DNA of each accession was extracted from young leaf tissues for genotyping using the DNAsecure Plant Kit (TIANGEN). A CottonSNP80K array containing 77,774 SNPs (Cai et al., 2017), which was recently developed based on the sequencing of “TM-1” (Zhang T. Z. et al., 2015) and the re-sequencing of 100 different cultivars in Upland cotton, with 5 × coverage on an average (Fang et al., 2017), were applied to genotype the 169 accessions. The image files were saved and analyzed using the GenomeStudio Genotyping Module (v1.9.4, Illumina). All 77,774 SNPs corresponded to the three separate signal clusters, AA, AB, and BB. However, from an evolutionary point of view, the polyploid cotton originated from an interspecific hybridization event between A- and D-genome diploid species around 1–2 million years ago, and the two extant progenitor relatives diverged from a common ancestor around 5–10 million years ago (Wendel and Cronn, 2003). In addition, Upland cotton is a type of cross-pollinated allotetraploid crop with a 10–15% natural hybridization rate. Thus, some SNPs in Upland cotton could contain five genotypes (AAAA, AAAB, AABB, ABBB, and BBBB). When these genotyping signals gather > 3 clusters, the automatic SNP calling can produce errors; therefore, we confirmed the genotypes of these loci using a manual adjustment method as described by Cai et al. (2017). Thus, a more accurate clustering file was produced to improve the genotyping efficiency levels for the samples.

### Population structure and LD estimation

Only SNPs with minor allele frequencies ≥0.05 and integrities ≥50% were used for population structure and LD analyses. The population structure was assessed using ADMIXTURE software (Alexander et al., 2009). To explore the population structure of the tested accessions, the number of genetic clusters (k) was predefined as 2–10. This analysis provided the maximum likelihood estimates of the proportion of each sample derived from each of the k sub-populations, and the corresponding Q-matrix was obtained for the subsequent GWAS. To determine the mapping resolution for GWAS, an LD analysis was performed for Upland cotton accessions. Pair-wise LD values between markers were calculated as the squared correlation coefficient (*r*^2^) of alleles using the GAPIT software (Lipka et al., 2012).

### GWAS

The GWAS was performed using six models, including three single-locus models: GLM (Bradbury et al., 2007), MLM (Bradbury et al., 2007), and compressed mixed linear model [CMLM; (Zhang et al., 2010)], and three multi-locus models: mrMLM (Wang et al., 2016), FASTmrEMMA (Wen et al., 2017), and ISIS EM-BLASSO (Tamba et al., 2017). In short, the GLM corrects only the population structure; the MLM corrects both population structure and kinship relationship among individuals; and the CMLM is equivalent to the MLM when individuals are clustered into groups based on kinship and the ratio of polygenic to residual variances is fixed by genome scanning. The three multi-locus models include two steps. The first step is to select all the potentially associated SNPs. In the next step, all the selected SNPs are included into one model, then their effects are estimated by empirical Bayes, and finally all the non-zero effects are further evaluated using the likelihood ratio test. FASTmrEMMA whitens the covariance matrix of the polygenic matrix K and environmental noise. In ISIS EM-BLASSO, an iterative modified sure independence screening along with SCAD algorithm was used to select potentially associated SNPs. In the three single-locus GWASs, significant levels of marker-trait association were set at an adjusted *P*-value of 1/n, after the Bonferroni correction (Cai et al., 2017; Sun et al., 2017), where n was the total number of SNPs used in GWAS. The Manhattan plots were drawn using the R package qqman (Turner, 2014). In the three multi-locus GWASs, the critical *P*-values were set at 0.01, 0.005, and 0.01 for mrMLM, FASTmrEMMA, and ISIS EM-BLASSO, respectively, in the first step. In the second step, all the critical LOD scores for significance were set at 3.0. The SNPs that met the above standards were identified as significant trait-associated QTNs.

### Identification of candidate genes

The R software package “LDheatmap” was used to determine the LD heatmaps surrounding the significant trait-associated QTNs. Based on the *G. hirsutum* “TM-1” genome (Zhang T. Z. et al., 2015), the genes within the LD decay distance on either side of the significant trait-associated SNPs were mined. To investigate the functions of these genes, RNA-seq datasets with two biological repetitions of 12 vegetative and reproductive tissues (root, stem, leaf, ovules from −3, −1, 0, 1, and 3 days post-anthesis, and fibers from 5, 10, 20, and 25 days post-anthesis) of *G. hirsutum* “TM-1,” were downloaded from the NCBI SRA database under accession code PRJNA248163 (http://www.ncbi.nlm.nih.gov/sra/?term=PRJNA248163; Zhang T. Z. et al., 2015). Normalized fragments per kilobase of transcript per million fragments mapped (FPKM) values were calculated to indicate the expression levels of these genes. The average of the two biological replicates was recorded as the final FPKM value. A heatmap of the expression patterns—based on FPKM values—of genes was created using Mev 4.9 (Saeed et al., 2003). Further gene annotations were performed from several databases for non-redundant protein sequences (ftp://ftp.ncbi.nih.gov/blast/db/FASTA; Altschul et al., 1997), gene ontology (http://www.geneontology.org; Ashburner et al., 2000), Cluster of Orthologous Groups of proteins (http://www.ncbi.nlm.nih.gov/COG; Tatusov et al., 2000), and the Kyoto Encyclopedia of Genes and Genomes (ftp://ftp.genome.jp/pub/kegg/; Kanehisa et al., 2004).

## Results

### Phenotypic variations in fiber quality traits

**Figure 1:**
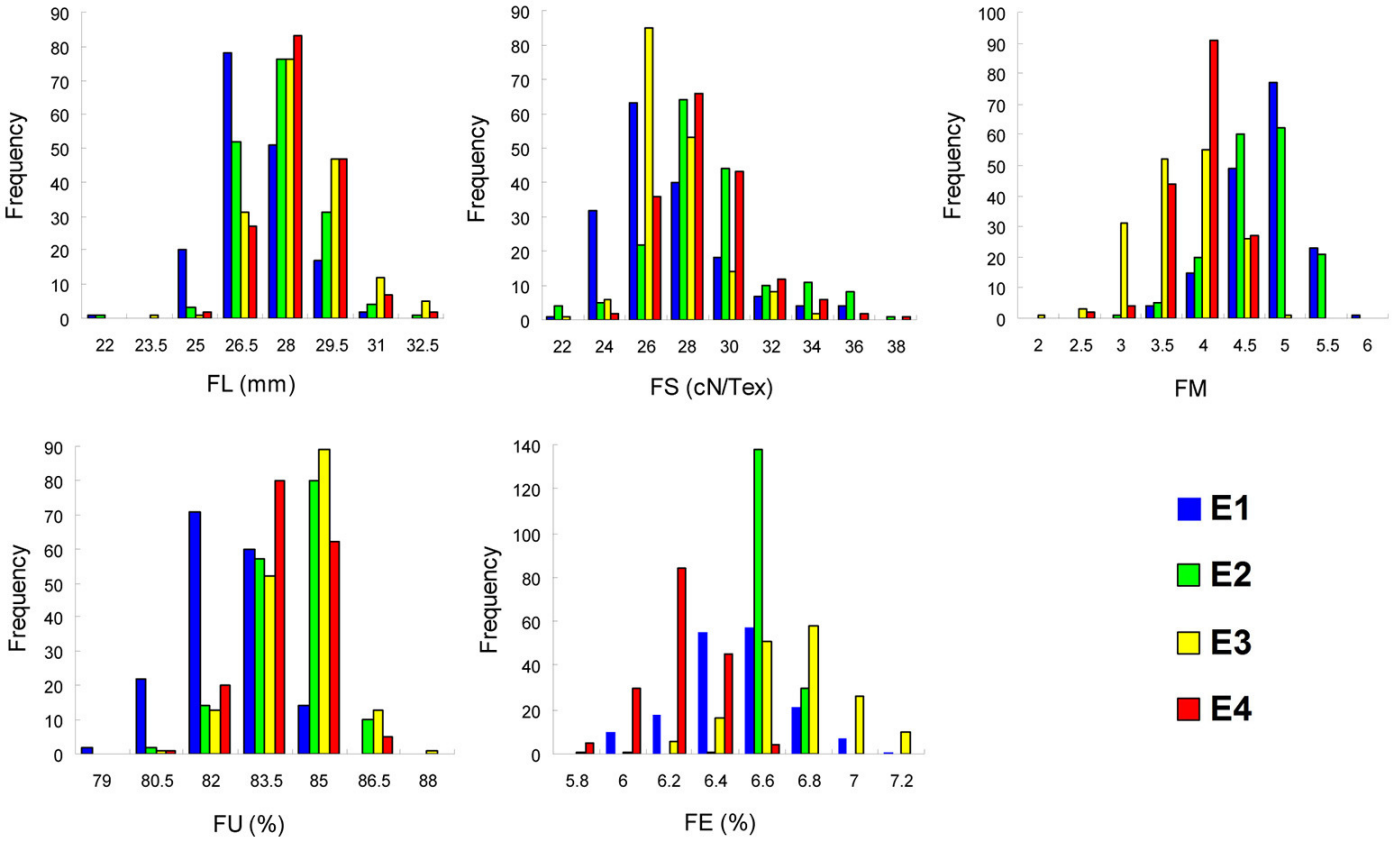
Frequency of the five fiber quality traits in 169 Upland cotton accessions. FL, fiber length; FS, fiber strength; FM, fiber micronaire; FU, fiber uniformity; FE, fiber elongation; E1, E2, E3, and E4 indicate four environments: 2012 Xinxiang, 2013 Xinxiang, 2012 Shihezi, and 2013 Shihezi, respectively.

Phenotypic values for five fiber quality traits of the 169 accessions in four environments (Supplementary Table S2) were used for the variation analysis. The phenotypic evaluation revealed a broad variation range among accessions. Descriptive statistics of phenotypic variation for the five fiber quality traits are listed in Table 1. The mean FL were 27.90, 28.52, 29.23, and 29.08 mm, respectively, in the four experiments. The minimum FL was 22.43 mm in E2, and the maximum FL was 34.48 mm in E3. Analogously, the other four traits of FS, FM, FU, and FE, exhibited values in the range of 23.40–39.90 cN/Tex, 2.10–6.03, 78.10–88.90%, and 5.70–7.50%, with means of 29.03 cN/Tex, 4.53, 84.53, and 6.59%, respectively. The CV ranges for FL, FS, FM, FU, and FE in the four environments were 4.69–5.40%, 6.85–9.52%, 8.87–15.73%, 1.34–1.74%, and 0.91–3.88%, respectively, and the average CVs for the same were 4.96, 8.59, 11.18, 1.52, and 2.81%, respectively. These data indicated different degrees of diversity in fiber quality traits in the natural population. The frequency distributions of the phenotypes (Figure 1) showed that the fiber quality traits exhibited the genetic characteristics of quantitative traits with continuous distributions across different environments. Furthermore, some of the traits exhibited multimodal or partial distributions, suggesting that the main effect genes/QTNs related to the target traits could exist in cotton genome.

**Table 1:** Descriptive statistics of phenotypic values of five fiber quality traits in four environments.

**Trait^a^**	**Env^b^**	**Min**	**Max**	**Average**	**Std**	**CV (%)**
FL (mm)	E1	23.18	31.32	27.90	1.36	4.86
	E2	22.43	33.06	28.52	1.39	4.87
	E3	24.20	34.48	29.23	1.58	5.40
	E4	24.91	34.40	29.08	1.36	4.69
FS (cN/Tex)	E1	23.80	37.80	28.16	2.68	9.52
	E2	23.50	38.70	30.14	2.82	9.35
	E3	23.40	35.20	28.23	1.93	6.85
	E4	24.30	39.90	29.58	2.56	8.66
FM	E1	3.67	6.00	5.05	0.45	8.87
	E2	3.38	5.84	4.96	0.46	9.19
	E3	2.10	6.03	3.93	0.62	15.73
	E4	2.59	5.21	4.17	0.46	10.92
FU (%)	E1	79.50	86.15	83.28	1.20	1.45
	E2	81.20	87.70	85.08	1.14	1.34
	E3	78.10	88.30	85.12	1.48	1.74
	E4	80.90	88.90	84.64	1.30	1.54
FE (%)	E1	6.00	7.35	6.57	0.23	3.56
	E2	6.50	6.90	6.71	0.06	0.91
	E3	5.80	7.50	6.80	0.26	3.88
	E4	5.70	6.80	6.29	0.18	2.90

a *FL, fiber length; FS, fiber strength; FM, fiber micronaire; FU, fiber uniformity; FE, fiber elongation*.

b *E1, E2, E3, and E4 indicate four environments: 2012 Xinxiang, 2013 Xinxiang, 2012 Shihezi, and 2013 Shihezi, respectively*.

### Characteristics of polymorphic SNPs

The genotypes of 169 accessions were examined using Illumina GenomeStudio software. Only the SNPs with minor allele frequencies ≥0.05, and integrities ≥50% in the population, were used for screening polymorphic loci. Thus, 53,848 high-quality SNPs were obtained out of 77,774. Their characteristics are summarized in Table 2 and Supplementary Figure S1. These SNPs were not evenly distributed across the *G. hirsutum* genome, and there were 28,454 and 25,394 SNPs in the A and D subgenomes, respectively. The average marker density was approximately one SNP per 38.02 kb. In the A subgenome, chromosome A6 had the most markers (2,982), with a marker density of one SNP per 34.60 kb, and A4 had the least markers (1,050), with a marker density of one SNP per 59.92 kb. In the D subgenome, chromosome D6 had the most markers (3,128), with a marker density of one SNP per 20.55 kb, and D4 had the least markers (1,040), with a marker density of one SNP per 49.48 kb. The polymorphism information content values ranged from 0.255 to 0.309 among chromosomes, and the mean polymorphism information content values of the A and D subgenomes were 0.285 and 0.284, respectively.

**Table 2:** Summary of the SNPs in 26 chromosomes of *Gossypium hirsutum*.

**Chr**.	**Chr. size (kb)**	**No. of SNPs**	**SNP density(kb/SNP)**	**Polymorphism information content value**
A1	99884.70	2371	42.13	0.301
A2	83447.91	1392	59.95	0.283
A3	100263.00	1744	57.49	0.277
A4	62913.77	1050	59.92	0.284
A5	92047.02	2575	35.75	0.300
A6	103170.40	2982	34.60	0.294
A7	78251.02	2125	36.82	0.290
A8	103626.30	2870	36.11	0.281
A9	74999.93	2439	30.75	0.277
A10	100866.6	2037	49.52	0.274
A11	93316.19	1915	48.73	0.280
A12	87484.87	2051	42.65	0.283
A13	83159.57	2903	28.65	0.285
D1	61456.01	1860	33.04	0.284
D2	67284.55	2371	28.38	0.307
D3	46690.66	1394	33.49	0.276
D4	51454.13	1040	49.48	0.282
D5	61933.05	1595	38.83	0.286
D6	64294.64	3128	20.55	0.275
D7	55312.61	2708	20.43	0.300
D8	65894.14	2273	28.99	0.309
D9	50995.44	2227	22.90	0.255
D10	63374.67	1734	36.55	0.290
D11	66087.77	1408	46.94	0.274
D12	59109.84	1968	30.04	0.273
D13	60534.30	1688	35.86	0.280

### Population structure and LD

**Figure 2:**
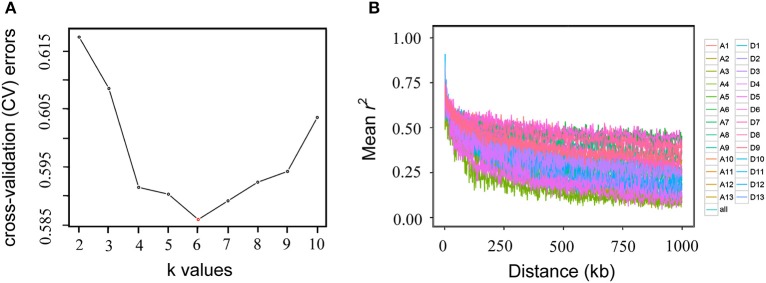
Population structure **(A)** and linkage disequilibrium decay **(B)** of 169 Upland cotton accessions. The accessions were divided into six sub-populations (the minimum number of cross-validation errors occurred when *k* = 6). Genome-wide average linkage disequilibrium decay was estimated in each of the 26 chromosomes and in all chromosomes.

To estimate the number of sub-populations in the population of 169 Upland cotton accessions, a population structure analysis was performed using the 53,848 SNPs. The results indicated that the minimum number of cross-validation errors was *k* = 6, which was thus determined to be the optimum k; and the testing accessions could be separated into six sub-populations (Figure 2A). The varietal population in this study was considered to be not highly structured and could be used for further association mapping. Thus, the corresponding Q-matrix from *k* = 6 was obtained for the subsequent GWAS. An LD analysis showed that the average LD decay distance for each of the 26 chromosomes ranged from 38.56 to 669.65 kb, and the average LD decay distance of all of the chromosomes (i.e., Upland cotton genome) was estimated to be 444.99 kb, with half of the maximum of mean *r*^2^-values (Figure 2B).

### GWAS for fiber quality traits

Three single-locus GWAS models: GLM, MLM, and CMLM, and three multi-locus GWAS models: mrMLM, FASTmrEMMA, and ISIS EM-BLASSO, were used to identify the marker–trait associations. In single-locus GWAS, the SNPs with –log_10_*P*≥4.73 (*P* = 1/53,848) were regarded as significant trait-associated SNPs. In multi-locus GWAS, the SNPs with LOD scores greater than 3.0 were regarded as significant trait-associated SNPs. Based on these criteria, 342 QTNs for fiber quality traits were detected using the values of individual environments (including BLUP) and the six models (Supplementary Table S3). To obtain reliable results, only the QTNs simultaneously detected in at least two environments, or by at least two models (either single-locus or multi-locus), were displayed. Finally, 84 QTNs controlling fiber quality traits were obtained (Table 3).

**Table 3:** Significant fiber quality trait-associated QTNs simultaneously detected in at least two environments or by at least two models.

**Trait^a^**	**SNP**	**Position (bp)**	**Alleles**	**Chr**.	**Single-locus GWAS**			**Env^d^**	**Multi-locus GWAS**			**Env^d^**
					**–log10*P***	***R*^2^ (%)^b^**	**Model^c^**		**LOD**	***R*^2^ (%)^b^**	**Model^c^**	
FL	TM10103	3462099	T/G	A5	5.10–5.41	12.02–13.71	G	E2, E3, Blup				
	TM10107	3488471	A/G	A5	4.82–6.71	14.02–16.67	G, M, C	E2, E3, Blup				
	TM10110	3505884	C/G	A5	5.09–5.42	11.76–13.47	G	E2, E3, Blup				
	TM10764	15474110	A/G	A5	4.85–4.85	14.69–14.69	M, C	Blup				
	TM39339	81827835	T/A	A11	5.16–5.22	12.19–13.28	G	E2, Blup				
	TM119	2771681	T/C	A1					3.36–4.06	7.81–10.32	MR, I	E1
	TM3930	3420685	T/C	A2					5.62–6.50	10.18–11.20	MR, I	E1
	TM4397	13758183	A/G	A2					6.62–8.35	7.49–10.46	F, MR, I	E2
	TM10319	5874999	A/C	A5					3.25–3.26	7.01–10.98	MR, I	E1
	TM10453	8801892	A/G	A5					4.69–6.05	5.66–10.20	MR, I	E1, E2
	TM10454	8829389	T/C	A5					6.70–7.87	9.33–9.56	I	E3, Blup
	TM10976	21365948	A/G	A5					4.55–4.71	17.78–23.57	MR, I	E4
	TM18271	99455219	T/C	A6					3,79–6.14	5.47–6.56	F, I	E3
	TM19208	14663920	T/G	A7					3.17–3.92	13.87–14.71	MR, I	E2
	TM27227	60265814	A/G	A8					3.73–3.78	11.70–12.84	I	E3, E4
	TM28899	77587957	A/G	A8					5.06–6.10	10.45–15.84	MR, I	E1
	TM31735	41160852	T/C	A9					4.06–6.05	3.33–5.15	F, I	E1, Blup
	TM33839	1954843	A/G	A10					3.38–4.83	5.42–11.24	MR, I	E1, Blup
	TM37371	13595750	A/G	A11					4.70–6.37	3.14–4.03	MR, I	E2
	TM42899	81101484	T/A	A12					4.69–5.08	5.07–5.35	MR, I	E1
	TM47849	1787530	T/C	D1					3.42–6.08	3.35–7.98	F	E1, Blup
	TM57343	16937262	A/G	D5					5.14–6.56	5.34–8.33	MR, I	E3
	TM58061	32206837	A/C	D5					3.78–4.92	4.43–6.03	MR, I	E2
	TM58758	59288520	T/C	D5					3.25–5.70	6.30–12.46	MR, I	E1, E3
	TM75008	58123221	T/C	D10					4.36–6.86	5.50–6.46	F, I	Blup
	TM75026	58453007	A/G	D10					3.33–4.19	4.17–5.60	F, MR, I	E3
	TM81924	55032877	A/C	D13					3.33–4.95	5.70–8.16	MR, I	E2
	TM57840	30021662	A/G	D5	4.88	10.35	G	E2	8.45–10.39	28.29–36.66	MR, I	E2
	TM80185	3106437	A/G	D13	4.96–5.42	13.08–14.46	G	E1, E3	3.28–6.88	3.94–8.88	F, I	E1, Blup
FS	TM10764	15474110	A/G	A5	4.94–6.26	10.48–15.16	G, M, C	E1, E2, E3, Blup				
	TM14418	30941574	T/C	A6	4.80–4.85	7.56–9.05	G	E1, E3				
	TM14424	31197620	T/C	A6	4.85–4.96	7.71–9.24	G	E1, E3				
	TM20073	28183664	T/G	A7	5.72–5.82	10.83–11.65	G	E3, Blup				
	TM21123	70595913	A/G	A7	4.83–5.30	8.61–8.67	G	E4, Blup				
	TM5639	80304252	T/C	A2					3.24–4.95	10.82–25.24	MR, I	E4
	TM10540	11387213	T/G	A5					3.19–7.74	5.03–15.89	MR, I	E2, Blup
	TM29912	101941614	T/A	A8					3.10–5.43	2.67–3.93	F, I	E3, Blup
	TM33273	65822047	A/C	A9					5.44–5.75	17.75–22.20	MR, I	E2
	TM42806	78617984	A/G	A12					3.23–3.52	11.50–12.06	MR, I	E2
	TM47849	1787530	T/C	D1					4.09–5.33	1.37–6.90	F, I	E2, E3, Blup
	TM57401	18161586	A/G	D5					5.48–8.15	8.96–10.25	MR, I	E1
	TM58758	59288520	T/C	D5					4.38–6.25	3.85–19.66	MR, I	E2, E3, Blup
	TM58839	61435904	T/G	D5					3.03–4.22	3.13–7.34	F, I	E1, Blup
	TM72234	38761458	A/G	D9					3.64–4.23	10.77–15.12	MR, I	E4
	TM74995	57945654	A/T	D10					4.21–4.23	14.26–19.43	MR, I	E4
	TM75026	58453007	A/G	D10					3.64–5.53	3.94–7.84	I	E3, Blup
	TM1386	41010954	T/C	A1	5.49–5.59	9.30–10.14	G	E1, Blup	5.21	23.95	I	E2
	TM5421	75968294	A/G	A2	4.87	10.20	G	E4	4.27–5.68	8.46–9.59	MR, F, I	E4
	TM14462	32121709	T/C	A6	4.83–5.68	10.27–11.64	G	E1, E2, Blup	5.03	6.32	I	E2
	TM21135	70682969	A/G	A7	5.02	10.09	G	E3	5.27–5.63	12.14–20.86	MR, I	E3
	TM79685	53877369	T/G	D12	4.82	8.81	G	E1	5.40	9.23	I	E1
FM	TM10764	15474110	A/G	A5	4.75–5.35	3.49–12.24	G, M, C	E2, Blup				
	TM18615	3643524	T/C	A7	5.04–5.80	10.74–12.04	G, M, C	E1				
	TM22010	5162186	A/T	A8					3.20–5.33	4.71–8.20	F, MR, I	E3
	TM33781	65693	A/G	A10					3.48–7.61	4.75–10.54	MR, I	E1, Blup
	TM42632	75299391	T/C	A12					3.02–3.39	3.12–3.94	F, I	E1
	TM55481	2866176	A/G	D4					3.00–5.66	0.96–3.34	MR, I	E1, E2, Blup
	TM57773	27544038	A/G	D5					3.30–4.56	2.93–3.96	I	E1, Blup
	TM18616	3646710	A/C	A7	5.11–5.52	10.94–12.16	G, M, C	E1	7.55–8.78	22.06–39.50	MR, I	E1, Blup
	TM19501	21060224	A/G	A7	4.84	11.05	G	E2	3.50	13.06	MR	E2
	TM54735	30908501	T/C	D3	4.76	11.33	G	Blup	4.00–9.64	5.70–16.76	F, MR, I	E3, Blup
	TM79518	51416454	T/G	D12	4.89–5.64	11.79–12.72	G, M, C	E3, Blup	5.44–8.17	27.75–53.97	MR, I	E3, Blup
FU	TM41077	26645691	G/C	A12	4.75–4.85	11.38–11.64	G	E1, Blup				
	TM18205	98260650	T/C	A6					3.36–4.77	7.64–10.52	MR, I	E1
	TM19379	18309921	T/C	A7					3.67–6.80	2.13–3.57	I	E3, Blup
	TM43826	15282624	A/G	A13					3.90–5.05	8.07–8.57	MR, I	Blup
	TM51438	21650323	A/G	D2					3.79–5.63	8.19–13.93	MR, I	Blup
	TM57831	29951748	A/G	D5					3.21–5.27	5.68–13.73	MR, I	Blup
	TM58758	59288520	T/C	D5					5.47–6.05	17.07–17.26	I	E2, E3
	TM67147	4674102	T/C	D8					3.07–3.13	9.82–15.67	MR, I	E1
	TM74995	57945654	A/T	D10					4.21–8.56	14.26–24.32	MR, I	E4, Blup
	TM11317	28285041	A/G	A5	5.09	12.82	G	Blup	4.70	7.79	F	Blup
	TM77489	3329594	T/C	D12	4.88	13.36	G	Blup	3.74–4.13	5.29–6.66	F	E1, Blup
	TM81448	45426771	C/G	D13	4.76–6.35	10.28–14.53	G, M, C	E4, Blup	7.85	26.18	MR	E4
FE	TM13701	2630501	T/C	A6					4.22–4.47	20.05–20.68	MR, I	Blup
	TM37254	7081938	A/G	A11					4.23–4.74	6.05–6.06	F, I	E2
	TM42798	78429684	C/G	A12					3.39–5.30	4.81–7.21	F	E1, E3
	TM43034	84964849	A/C	A12					3.66–5.80	15.22–15.30	MR, I	E1
	TM43327	3481958	A/G	A13					3.38–4.24	9.21–10.04	MR, I	E2
	TM48070	5563241	A/G	D1					3.25–4.10	3.59–5.91	MR, I	Blup
	TM63323	4045155	T/C	D7					4.35–5.89	32.47–34.06	MR, I	E4
	TM74999	57965498	A/G	D10					4.17–4.77	7.41–8.20	MR, F	E4
	TM77062	58739941	A/C	D11					4.13–4.34	17.94–21.38	MR, I	E2
	TM47772	723752	T/C	D1	5.68	14.55	G	E3	3.34–7.75	4.54–19.68	MR, F, I	E1, E3

a *FL, fiber length; FS, fiber strength; FM, fiber micronaire; FU, fiber uniformity; FE, fiber elongation*.

b *R^2^ (%) means phenotypic variation explained by marker*.

c *G, M, C, MR, F, and I represent GLM, MLM, CMLM, mrMLM, FASTmrEMMA, and ISIS EM-BLASSO, respectively*.

d *E1, E2, E3, E4, and Blup indicate 2012 Xinxiang, 2013 Xinxiang, 2012 Shihezi, 2013 Shihezi, and best linear unbiased predictor, respectively*.

Based on FL, 29 QTNs were detected. Five SNPs, including TM10103, TM10107, TM10110, TM10764, and TM39339, located on A5 and A11, were significantly associated with the E2, E3, and/or BLUP values by a single-locus GWAS, and this explained 11.76–16.67% of the phenotypic variations. 22 SNPs, including TM119, TM3930, and TM4397, located on A1, A2, A5, A6, A7, A8, A9, A10, A11, A12, D1, D5, D10, and D13, were significantly associated with the E1, E2, E3, E4, and/or BLUP values by a multi-locus GWAS, and this explained 3.14–23.57% of the phenotypic variations. Two SNPs, TM57840, and TM80185, respectively located on D5 and D13, were significantly associated with the E1, E2, E3, and/or BLUP values by both single-locus and multi-locus GWAS, which explained 10.35–14.46% of phenotypic variations in single-locus GWAS and 3.94–36.66% in multi-locus GWAS.

Based on FS, 22 QTNs were detected. Five SNPs, including TM10764, TM14418, TM14424, TM20073, and TM21123, located on A5, A6, and A7, were significantly associated with the E1, E2, E3, E4, and/or BLUP values by a single-locus GWAS, thus explaining 7.56–15.16% of the phenotypic variations. Additionally, 12 SNPs, including TM5639, TM10540, and TM29912, located on A2, A5, A8, A9, A12, D1, D5, D9, and D10, were significantly associated with the E1, E2, E3, E4, and/or BLUP values by a multi-locus GWAS, thus explaining 1.37–25.24% of the phenotypic variations. Five SNPs, including TM1386, TM5421, TM14462, TM21135, and TM79685, respectively located on A1, A2, A6, A7, and D12, were significantly associated with the E1, E2, E3, E4, and/or BLUP values by both single-locus and multi-locus GWASs, and this explained 8.81–11.64% of the phenotypic variations in the single-locus GWAS and 6.32–23.95% in the multi-locus GWAS.

Based on FM, 11 QTNs were detected. Two SNPs, TM10764 and TM18615, respectively located on A5 and A7, were significantly associated with the E1, E2 and/or BLUP values by a single-locus GWAS, and this explained 3.49–12.24% and 10.74–12.04% of the phenotypic variations. Five SNPs, TM22010, TM33781, TM42632, TM55481, and TM57773, located on A8, A10, A12, D4, and D5, respectively, were significantly associated with the E1, E2, E3, and/or BLUP values by a multi-locus GWAS, thus explaining 0.96–10.54% of the phenotypic variations. Four SNPs, TM18616, TM19501, TM54735, and TM79518, located on A7, D3, and D12, were significantly associated with the E1, E2, E3, and/or BLUP values by both single-locus and multi-locus GWASs, thus explaining the phenotypic variations of 10.94–12.72% in the single-locus GWAS and 5.70–53.97% in the multi-locus GWAS.

Based on FU, 12 QTNs were detected. One SNP, TM41077, located on A12, was significantly associated with the E1 and BLUP values by a single-locus GWAS, and this explained 11.38–11.64% of the phenotypic variations. Eight SNPs, including TM18205, TM19379, and TM43826, located on A6, A7, A13, D2, D5, D8, and D10, were significantly associated with the E1, E2, E3, E4, and/or BLUP values by a multi-locus GWAS, thus explaining 2.13–24.32% of the phenotypic variations. Three SNPs, TM11317, TM77489, and TM81448, respectively located on A5, D12, and D13, were significantly associated with the E1, E4, and/or BLUP values by both single-locus and multi-locus GWASs, thus explaining the phenotypic variations of 10.28–14.53% in the single-locus GWAS and 5.29–26.18% in the multi-locus GWAS.

Based on FE, 10 QTNs were detected. Nine SNPs, including TM13701, TM37254, and TM42798,r located on A6, A11, A12, A13, D1, D7, D10, and D11, were significantly associated with the E1, E2, E3, E4, and/or BLUP values by a multi-locus GWAS, thus explaining 3.59–34.06% of the phenotypic variations. One SNP, TM47772, located on D1, was significantly associated with the E1 and/or E3 values by both single-locus and multi-locus GWASs, thus explaining 14.55% of the phenotypic variations in the single-locus GWAS and 4.54–19.68% in the multi-locus GWAS.

### Identification and expression of candidate genes for fiber quality

**Figure 3:**
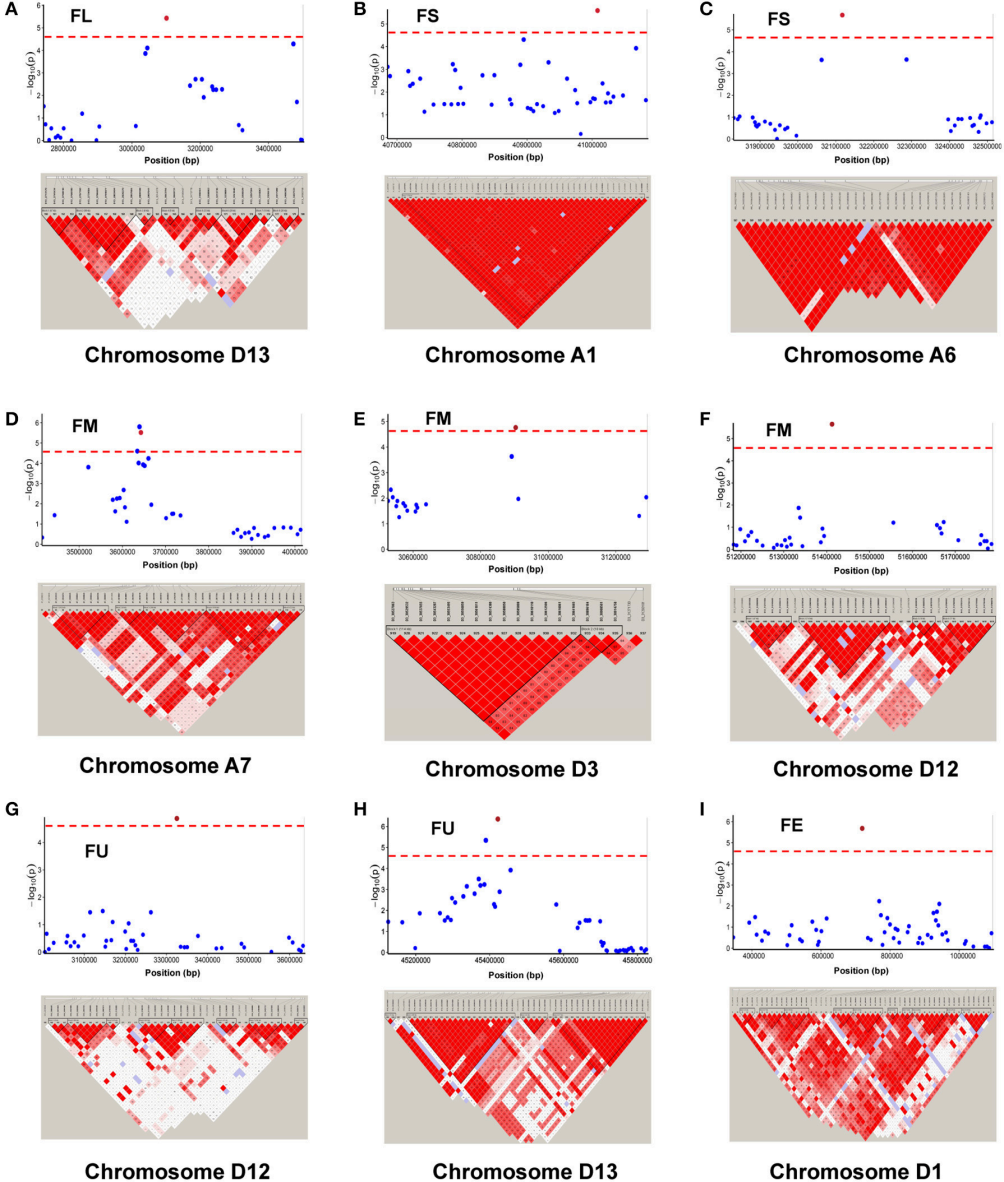
Genomic location of nine QTNs simultaneously detected in at least two environments, by both single-locus GWAS and multi-locus GWAS, and LD heatmaps surrounding nine QTNs for **(A)** fiber length (FL) on chromosome D13, **(B,C)** fiber strength (FS) on chromosomes A1 and A6, **(D–F)** fiber micronaire (FM) on chromosomes A7, D3, and D12, **(G,H)** fiber uniformity (FU) on chromosome D12 and D13, and **(I)** fiber elongation (FE, %) on chromosome D1.

**Figure 4:**
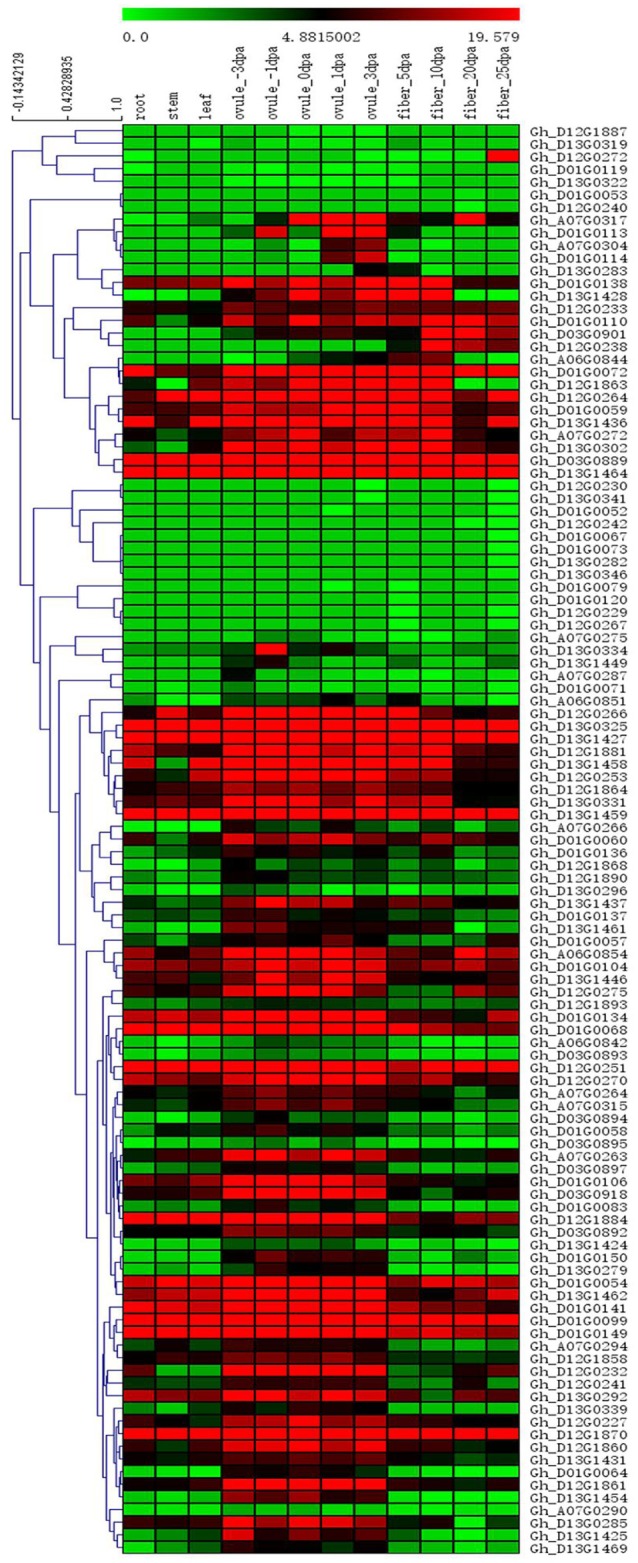
Heatmap of expression patterns of 113 genes with hierarchical clustering based on FPKM values. These genes presented higher expression levels in ovules and/or fiber during their developmental stages, while being less expressed in root, stem, and leaf. The values in the horizontal color bar are automatically generated in Mev 4.9 according to the FPKM values; red indicates high expression, and green indicates low expression.

**Figure 5:**
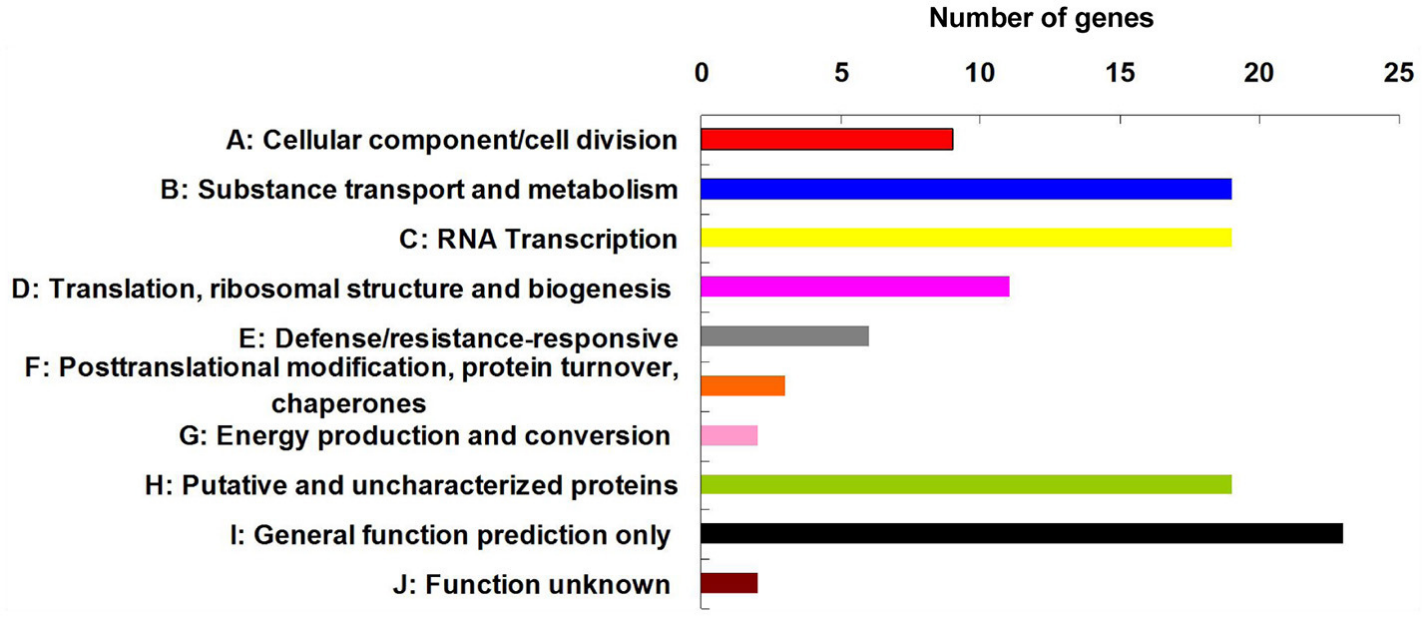
Functional classification of 113 candidate genes, which presented higher expression levels in ovules and/or fiber during the stages of their development, while being less expressed in root, stem, and leaf.

Among the 84 QTNs, nine QTNs—TM80185 (D13) associated with FL, TM1386 (A1) and TM14462 (A6) associated with FS, TM18616 (A7), TM54735 (D3), and TM79518 (D12) associated with FM, TM77489 (D12) and TM81448 (D13) associated with FU, and TM47772 (D1) associated with FE, were simultaneously detected in at least two environments, and by both single-locus and multi-locus GWASs (Supplementary Figures S2–S6), indicating that they were more stable. Considering the LD decay distance of the Upland cotton population used in this study, the regions within 400-kb on either side of the nine QTNs were used for the further identification of candidate genes. The LD analysis showed that a high LD level existed among the SNPs within 400-kb upstream and downstream of the nine QTNs in D13 (Figure 3A) for FL, A1 (Figure 3B) and A6 (Figure 3C) for FS, A7 (Figure 3D), D3 (Figure 3E), and D12 (Figure 3F) for FM, D12 (Figure 3G) and D13 (Figure 3H) for FU, and D1 (Figure 3I) for FE. Multiple LD blocks were included in almost all of the LD regions except those in A6 (Figure 3C). As a result, 455 genes were around the above nine QTNs. The normalized FPKM values of 455 genes, representing their expression levels, are displayed in Supplementary Table S4. To investigate which genes were responsible for fiber quality, only those genes that presented greater expression levels in ovules and/or fiber during their developmental stages, while being less expressed in root, stem, and leaf, were used for further functional analyses. Thus, 113 genes, marked in bold in Supplementary Table S4, were obtained. A heatmap of the expression patterns of these genes with hierarchical clustering based on FPKM values is shown in Figure 4. Considering that the five fiber quality traits are directly related to fiber development and are significantly positively correlated with each other, these genes were merged into a group for a systematic summary according to the functional annotation from the non-redundant protein, gene ontology, Cluster of Orthologous Groups of proteins, and the Kyoto Encyclopedia of Genes and Genomes analyses (Supplementary Table S5). These 113 genes could be classified into 10 categories (Figure 5), which include 9 in “Cellular component/cell division” (A), 19 in “Substance transport and metabolism” (B), 19 in “RNA Transcription” (C), 11 in “Translation, ribosomal structure and biogenesis” (D), 6 in “Defense/resistance-responsive” (E), 3 in “Post-translational modification, protein turnover, chaperones” (F), 2 in “Energy production and conversion” (G), 19 in “Putative and uncharacterized proteins” (H), 23 in “General function prediction only” (I), and 2 in “Function unknown” (J). Several promising candidate genes were found through further bioinformatics analyses. *Gh_D13G1461* is homologous to Arabidopsis *AT1G50660*, which is the predicted protein sequence for the *BRANCHLESS TRICHOMES* gene, a key positive regulator of trichome branching (Marks et al., 2009; Kasili et al., 2015). *Gh_D12G0232* is homologous to Arabidopsis *AT2G03500*, which encodes a nuclear localized member of the *MYB* family of transcriptional regulators. The *MYB* transcription factor plays a role in cotton fiber and trichome development (Machado et al., 2009). Cellulose is the main component of cotton fiber. *Gh_D01G0052* and *Gh_D12G0240* are both homologous with Arabidopsis *AT1G09790*, which is annotated as a COBRA-like protein 6 precursor. In *Arabidopsis thaliana*, the COBRA is involved in determining the orientation of cell expansion, playing an important role in cellulose deposition (Roudier et al., 2005). Thus, the four genes might be promising candidate genes for improving the fiber quality.

## Discussion

### Large numbers of high-quality SNPs ensure effective GWAS in cotton

Association mapping is a powerful tool in dissecting the genetic basis of plant complex traits. Prior to the availability of next-generation sequencing techniques; however, SSR markers were mainly used to detect molecular markers associated with the target traits. Due to a limited number of markers, the genetic basis of the quantitative traits could not be fully revealed at the genome-wide level. With the wide application of high-density genotyping platforms, the development of numerous SNPs makes it possible to perform GWASs of the genetic bases of complex traits. In cotton, the SNPs developed from next-generation sequencing methods, such as specific-locus amplified fragment sequencing and genotyping-by-sequencing, were used to perform GWASs for lint percentage (Su et al., 2016a), fiber quality (Islam et al., 2016; Su et al., 2016b), early maturity (Su et al., 2016c), and *Verticillium* wilt resistance (Li T. et al., 2017). Furthermore, the first commercial high-density CottonSNP63K array, developed from 13 different discovery sets that represent a diverse range of *G. hirsutum* germplasm, as well as five other species, provided a new resource for the genetic dissection of cotton's quantitative traits (Hulse-Kemp et al., 2015). Presently, based on the CottonSNP63K array, the GWASs have been performed to unravel the agronomically and economically important traits in cotton, including yield components, fiber quality, growth period, plant height, and stomatal conductance (Gapare et al., 2017; Huang et al., 2017; Sun et al., 2017). Compared with CottonSNP63K, the recently developed CottonSNP80K array is more useful for dissecting the genetic architecture of important traits in Upland cotton because the SNP loci in the array benefited from the whole-genome sequencing of *G. hirsutum* acc. TM-1 (Zhang T. Z. et al., 2015) and 1,372,195 intraspecific non-unique SNPs identified by the re-sequencing of *G. hirsutum* accessions (Fang et al., 2017). In addition, each SNP marker in the CottonSNP80K array is addressable, which avoids the disturbances caused by homeologous/paralogous genes. The diverse application tests indicate that CottonSNP80K played important roles in germplasm genotyping, varietal verification, functional genomics studies, and molecular breeding in cotton (Cai et al., 2017). In this study, 53,848 high-quality SNPs out of 77,774 from the CottonSNP80K array, accounting for 69.24% of all loci, were screened in our experimental accessions. The large number of high-quality SNPs will be very conducive to unravel the genetic architecture of the target traits through GWASs.

### Combining single- and multi-locus GWASs can improve the power and robustness of GWAS

With the development of molecular quantitative genetics, a large number of association mapping methods have emerged for the genetic dissection of complex traits in plants (Feng et al., 2016). However, the methods used in most of the previous studies are single-locus analysis approaches based on a fixed-SNP-effect mixed linear model under a polygenic background and population structure controls. These methods require a Bonferroni correction for multiple tests. To control the experimental error at a genome-wide level of 0.05, the significance level for each test should be adjusted by 0.05/n (n is the total number of SNPs). The use of stringent probability thresholds reduces the risk of accepting false positives but does not reduce the risk of rejecting true positives caused by setting the very high thresholds. Multi-locus models, such as Bayesian LASSO (Yi and Xu, 2008), penalized Logistic regression (Hoggart et al., 2008), adaptive mixed LASSO (Wang et al., 2010), and EBAYES LASSO (Wen et al., 2015), can improve the efficiency and accuracy of QTL detection in GWAS. An obvious advantage of these models is that no Bonferroni correction is required because of the multi-locus nature. In particular, several recently developed multi-locus models, including mrMLM (Wang et al., 2016), FASTmrEMMA (Wen et al., 2017), and LASSO (ISIS EM-BLASSO) (Tamba et al., 2017), have been demonstrated as having the highest power and accuracy levels for QTL detection when compared with some former methods. As the inheritance of quantitative traits is complex and the number of markers is several times larger than the sample sizes, it is necessary to simultaneously use multiple methods for GWAS. Several examples can be found in previous studies. Li H. G. et al. (2017) performed a GWAS to reveal the genetic control underlying the branch angle in rapeseed by simultaneously using a single-locus model, MLM, and a multi-locus model, mrMLM. As a result, more than 55% of the loci identified using mrMLM overlapped part or most of the region of those obtained using MLM. Misra et al. (2017) determined the genetic basis of cooked grain length and width in rice using four GWAS methods—EMMAX, mrMLM, FASTmrEMMA, and ISIS EM-BLASSO. Thus, employing integrated single-locus and multi-locus GWAS models led to the verification of the significance of the underlying target regions, GWi7.1 and GWi7.2, and simultaneously identified the novel candidate genes. In this study, using three single-locus and three multi-locus models, 342 significant QTNs were identified. More loci were identified using multi-locus models than using single-locus models, and 15 loci were simultaneously identified in both single-locus and multi-locus models (Supplementary Table S3). These findings demonstrated the reliability of association analysis consequences and the practicality of combining single-locus and multi-locus GWASs to improve the power and robustness of association analyses.

### Stable QTNs for fiber quality traits detected in our GWAS

The marker loci/QTLs that are detected across multiple populations, environments and/or mapping methods, are highly stable and can enhance the efficiency and accuracy of the MAS (Su et al., 2010; Li et al., 2013). In cotton, using linkage mapping, Jia et al. (2011) located five QTLs for boll weight and lint percentage that were stably expressed in several environments by two mapping methods. Li et al. (2012) identified two QTLs for the node of the first fruiting branch and its height by two mapping methods. Sun et al. (2012) identified two QTLs for FS, which were simultaneously detected in four environments. Cai et al. (2014) performed association mapping of fiber quality traits and identified 70 significantly associated marker loci, of which 36 and four coincided with previously reported QTLs identified using linkage and association mapping populations, respectively. Here, 342 QTNs significantly associated with the fiber quality traits were detected using the values of individual environments (including BLUPs) and the six models. However, to obtain reliable results, only the QTNs simultaneously detected in at least two environments or by at least two models were displayed, and thus, 84 QTNs controlling the fiber quality traits were obtained. Of them, 29 were for FL, 22 were for FS, 11 were for FM, 12 were for FU, and 10 were for FE. These QTNs are highly stable and can potentially be used in the MAS of target traits. Additionally, nine QTNs, TM80185 (D13) for FL, TM1386 (A1) and TM14462 (A6) for FS, TM18616 (A7), TM54735 (D3), and TM79518 (D12) for FM, TM77489 (D12) and TM81448 (D13) for FU, and TM47772 (D1) for FE, were simultaneously detected in at least two environments, and by both single-locus and multi-locus GWASs. These nine QTNs also exhibited high phenotypic contributions of more than 10% in either a single-locus or multi-locus GWAS. Therefore, they could be given priority for MAS in future breeding programs.

### Comparison of our GWAS with the results in previous studies

Presently, several QTLs/markers related to cotton fiber qualities have been identified using linkage mapping and association mapping in previous studies (Shen et al., 2005; Abdurakhmonov et al., 2008, 2009; Kantartzi and Stewart, 2008; An et al., 2010; Sun et al., 2012, 2017; Wang et al., 2013; Zhang et al., 2013; Cai et al., 2014; Qin et al., 2015; Islam et al., 2016; Li C. et al., 2016; Nie et al., 2016; Su et al., 2016b; Gapare et al., 2017; Huang et al., 2017; Iqbal and Rahman, 2017; Ma et al., 2017; Sethi et al., 2017; Tan et al., 2018). We compared the 342 QTNs detected in our GWAS (Supplementary Table S3) with SNPs and SSRs linked to/associated with QTLs for the same traits identified in previous studies by electronic PCR (e-PCR) based on their physical locations on the genome sequence (Zhang T. Z. et al., 2015). The markers linked to/associated with QTLs for the same traits that were located within the same region of ~400 kb, were regarded as the same loci. Thus, 12 QTNs detected in our GWAS corresponded to previously reported SNPs and SSRs detected based on linkage and/or association mapping (Table 4). Specifically, two QTNs for FL, TM58426 (D5) and TM72875 (D9), corresponded to BNL4047 (Sethi et al., 2017) and DPL0395 (Sun et al., 2012)/MGHES-55 (Iqbal and Rahman, 2017), respectively; five QTNs for FS, TM5639 (A2), TM21292 (A7), TM43422 (A13), TM63860 (D7), and TM74995 (D10), corresponded to HAU880 (Wang et al., 2013), i18340Gh/i44206Gh/i39753Gh/i02033Gh/i02034Gh/i02035Gh/i02037Gh/i49171Gh/i37604Gh (Sun et al., 2017), i30934Gh (Sun et al., 2017), BNL3854 (An et al., 2010), and TM74991 (Tan et al., 2018), respectively; one QTN for FM, TM52959 (D2), corresponded to NAU2353 (Sun et al., 2012); two QTNs for FU, TM72633 (D9) and TM74995 (D10), corresponded to MGHES-6 (Iqbal and Rahman, 2017) and TM74991 (Tan et al., 2018), respectively; five QTNs for FE, TM3939 (A2), TM56516 (D4), TM72628 (D9), TM74999 (D10), and TM80198 (D13), corresponded to BNL1434 (Kantartzi and Stewart, 2008; Sethi et al., 2017), i12839Gh (Sun et al., 2017), BNL1030 (Kantartzi and Stewart, 2008), TM74991 (Tan et al., 2018), and NAU2730 (Sun et al., 2012), respectively. The 15 QTNs controlling the fiber quality, which were simultaneously detected in different populations with different genetic backgrounds, can potentially be used in the MAS of target traits.

**Table 4:** 12 QTNs controlling fiber quality traits identified in both this and previous studies.

**Trait^a^**	**GWAS in this study**			**Previous studies**			
	**Maker associated**	**Chr**.	**Position (bp)**	**Marker linkaged/associated^b^**	**Chr**.	**Position (bp)**	**References**
FL	TM58426	D5	52167190	BNL4047 (AM)	D5	51715146~51715301	Sethi et al., 2017
	TM72875	D9	47994726	DPL0395 (LM), MGHES-55 (AM)	D9	48340706~48340931, 48074891~48075112	Sun et al., 2012; Iqbal and Rahman, 2017
FS	TM5639	A2	80304252	HAU880 (LM)	A2	80045222~80045391	Wang et al., 2013
	TM21292	A7	72067994	i18340Gh, i44206Gh, i39753Gh, i02033Gh, i02034Gh, i02035Gh, i02037Gh, i49171Gh, i37604Gh (AM)	A7	71993462~72249786	Sun et al., 2017
	TM43422	A13	5198708	i30934Gh (AM)	A13	5168143	Sun et al., 2017
	TM63860	D7	14495698	BNL3854 (LM)	D7	14236226~14236344	An et al., 2010
	TM74995	D10	57945654	TM74991 (LM)	D10	57899125	Tan et al., 2018
FM	TM52959	D2	60834004	NAU2353 (LM)	D2	60579477~60579638	Sun et al., 2012
FU	TM72633	D9	44334923	MGHES-6 (AM)	D9	44634167~44634349	Iqbal and Rahman, 2017
	TM74995	D10	57945654	TM74991 (LM)	D10	57899125	Tan et al., 2018
FE	TM3939	A2	3531460	BNL1434 (AM)	A2	3419328~3419575	Kantartzi and Stewart, 2008; Sethi et al., 2017
	TM56516	D4	47872954	i12839Gh (AM)	D4	47872770	Sun et al., 2017
	TM72628	D9	44115527	BNL1030 (AM)	D9	43992085~43992321	Kantartzi and Stewart, 2008
	TM74999	D10	57965498	TM74991 (LM)	D10	57899125	Tan et al., 2018
	TM80198	D13	3477308	NAU2730 (LM)	D13	3582661~3582860	Sun et al., 2012

a *FL, fiber length; FS, fiber strength; FM, fiber micronaire; FU, fiber uniformity; FE, fiber elongation*.

b *AM and LM mean association mapping and linkage mapping, respectively*.

### Candidate genes for fiber quality traits

The identification of stable marker loci/QTLs could provide useful information for MAS. Candidate gene analyses are necessary for further gene cloning and functional verifications. Some candidate genes related to cotton fiber quality have already been identified using the GWAS approach. Islam et al. (2016) identified candidate genes related to fiber quality by gene expression and amino acid substitution analysis and suggested that the *Gh_A07G2049* (*GhRBB1_A07*) gene is a candidate for superior fiber quality in Upland cotton. Sun et al. (2017) identified 19 promising candidate genes related to FL and FS, of which, *Gh_A07G1758* could play a key role in the formation of cotton fiber, while *Gh_D03G0294* and *Gh_D05G1451* could play different roles during fiber development. In the study of Su et al. (2016b), three potential candidate genes, *CotAD_22823, CotAD_22824*, and *CotAD_22825*, for FL were identified, and the two peak SNPs (rsDt7:25931998 and rsDt7:25932026) associated with FL were positioned within one of the introns of *CotAD_22823*. In this study, 455 candidate genes surrounding the nine QTNs, which were simultaneously detected in at least two environments, were identified by both single-locus and multi-locus GWASs. Of the 455 candidate genes, 113 were highly expressed in ovules and/or fiber during their development, while being less expressed in root, stem, and leaf, suggesting that these genes might potentially affect the formation and development of cotton fiber, and thus contribute to fiber quality. These genes were categorized based on their functional characteristics from several databases. We cannot accurately determine which genes are directly related to fiber quality based on the data of this study. However, the results will provide useful information for future works. Cotton fiber development shares many similarities with the trichomes of Arabidopsis leaves in cellular and genetic features (Serna and Martin, 2006). Further, bioinformatics analyses indicated that the four genes, *Gh_D13G1461, Gh_D12G0232, Gh_D01G0052*, and *Gh_D12G0240*, may be promising candidate genes for improving the fiber quality. However, the formation of cotton fiber is a complicated physiological and biochemical process that might involve a large number of structural, regulatory, and biochemical pathway-related genes. Therefore, the functions of many genes in cotton remain to be elucidated.

## Conclusion

This research reported the GWAS of fiber quality traits in Upland cotton based on a recently developed CottonSNP80K array. A total of 342 QTNs controlling the fiber quality traits were detected via three single-locus and three multi-locus models. Of these QTNs, 84 were simultaneously detected in at least two environments or by at least two models. Further, nine QTNs were simultaneously detected in at least two environments, and by both single- and multi-locus models. 12 QTNs corresponded to previously reported SNPs and SSRs. In total, 455 candidate genes were identified within 400-kb upstream and downstream of the above nine QTNs based on the genome sequence of Upland cotton. Among these genes, 113 might potentially affect the formation and development of cotton fiber and four might be promising candidate genes for improving fiber quality.

## Author contributions

CL designed the experiment and wrote the manuscript. QW provided the experimental materials. YF, RS, and YW performed the experiments. All authors commented on the manuscript.

### Conflict of interest statement

The authors declare that the research was conducted in the absence of any commercial or financial relationships that could be construed as a potential conflict of interest.

## Supplementary material

Supplementary Table S1 Names and ecological sources of the 169 Upland cotton accessions.

Supplementary Table S2 Phenotypic values for the fiber quality traits of the 169 accessions in four environments.

Supplementary Table S3 All 342 QTNs for fiber quality traits detected using the values of individual environments (including BLUP) and the six models.

Supplementary Table S4 Normalized fragments per kilobase of transcript per million fragments mapped values of the 455 candidate genes.

Supplementary Table S5 A systematic summary of the 113 candidate genes.

Supplementary Figure S1 Single nucleotide polymorphism distributions on the 26 chromosomes of Upland cotton.

Supplementary Figure S2 QTN, TM80185 (D13), associated with FL, was simultaneously detected in at least two environments, by both single-locus and multi-locus GWASs.

Supplementary Figure S3 QTNs, TM1386 (A1), and TM14462 (A6), associated with FS, were simultaneously detected in at least two environments, by both single-locus and multi-locus GWASs.

Supplementary Figure S4 QTNs, TM18616 (A7), TM54735 (D3), and TM79518 (D12), associated with FM, were simultaneously detected in at least two environments, by both single-locus and multi-locus GWASs.

Supplementary Figure S5 QTNs, TM77489 (D12), and TM81448 (D13), associated with FU, were simultaneously detected in at least two environments, by both single-locus and multi-locus GWASs.

Supplementary Figure S6 QTN, TM47772 (D1), associated with FE, was simultaneously detected in at least two environments, by both single-locus and multi-locus GWASs.

## Abbreviations

FASTmrEMMA: fast multi-locus random-SNP-effect EMMA
FE: Fiber elongation
FL: Fiber length
FM: Fiber micronaire
FS: Fiber strength
FU: Fiber uniformity
GLM: general linear model
GWAS: Genome-wide association study
ISIS EM-BLASSO: Iterative modified-Sure Independence Screening EM-Bayesian LASSO
LD: Linkage disequilibrium
MAS: Marker-assisted selection
MLM: Mixed linear model
mrMLM: multi-locus RMLM
QTLs: Quantitative trait loci
QTN: quantitative trait nucleotide
SNP: Single-nucleotide polymorphism
SSR: Simple sequence repeat.
