# Supplementary figures and images for: Single-Locus and Multi-Locus Genome-Wide Association Studies in the Genetic Dissection of Fiber Quality Traits in Upland Cotton (Gossypium hirsutum L.)
Source: Front Plant Sci. 2018 Aug 17;9:1083. doi: 10.3389/fpls.2018.01083 (PMC6109694; doi:10.3389/fpls.2018.01083)

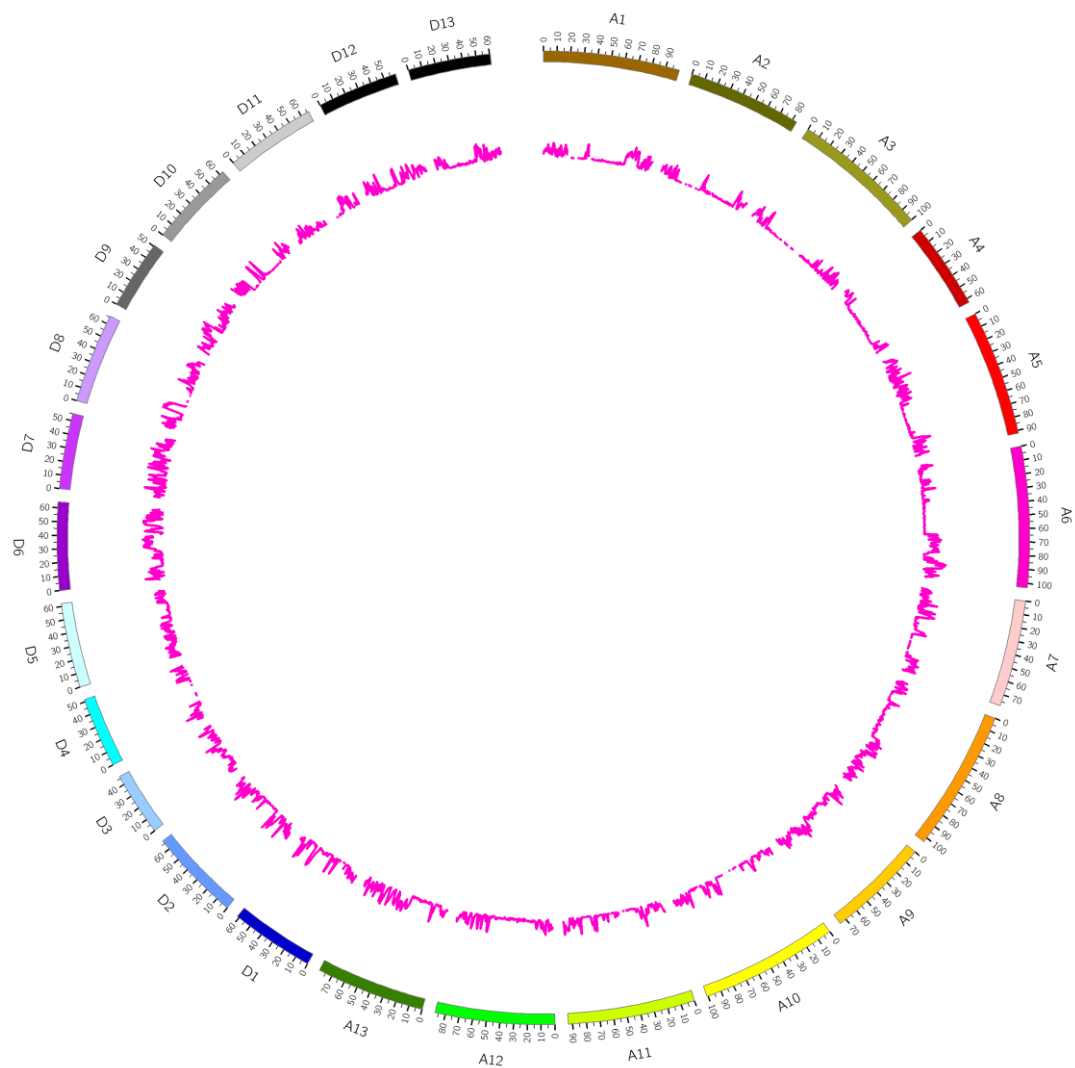

Supplement: Supplementary Figure S1 — Single nucleotide polymorphism distributions on the 26 chromosomes of Upland cotton. [file Data_Sheet_1.PDF]

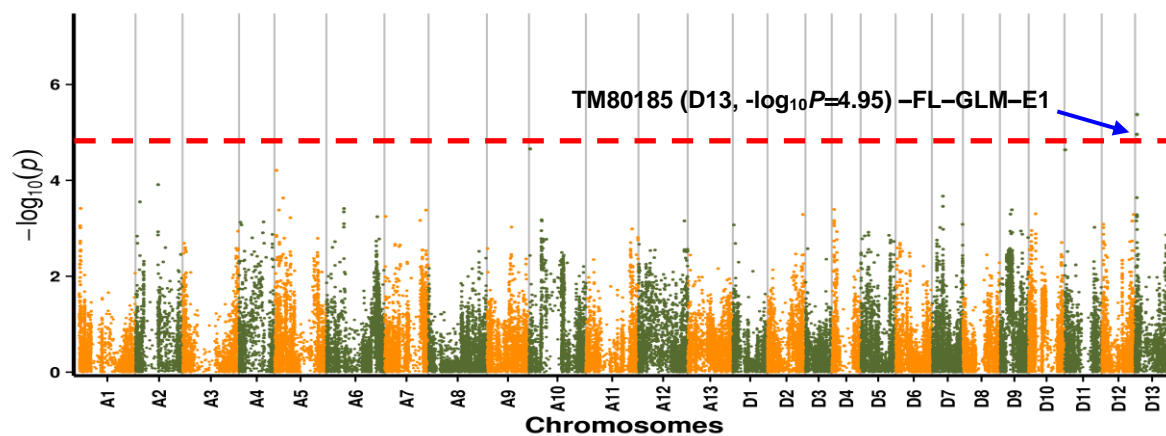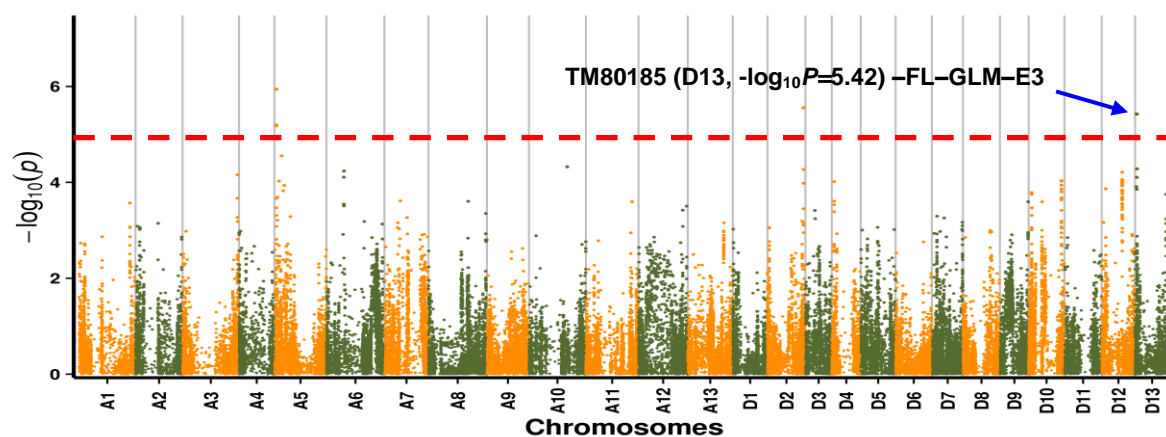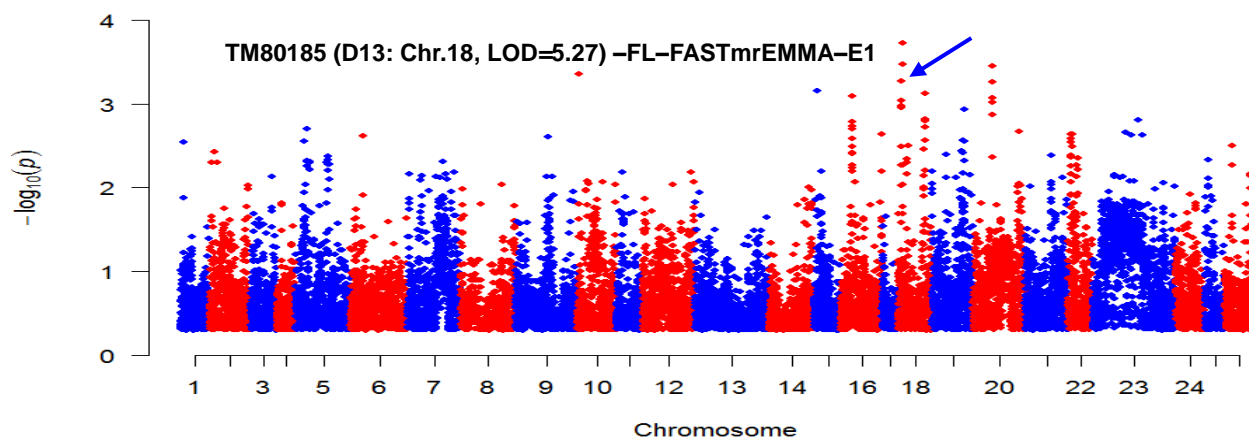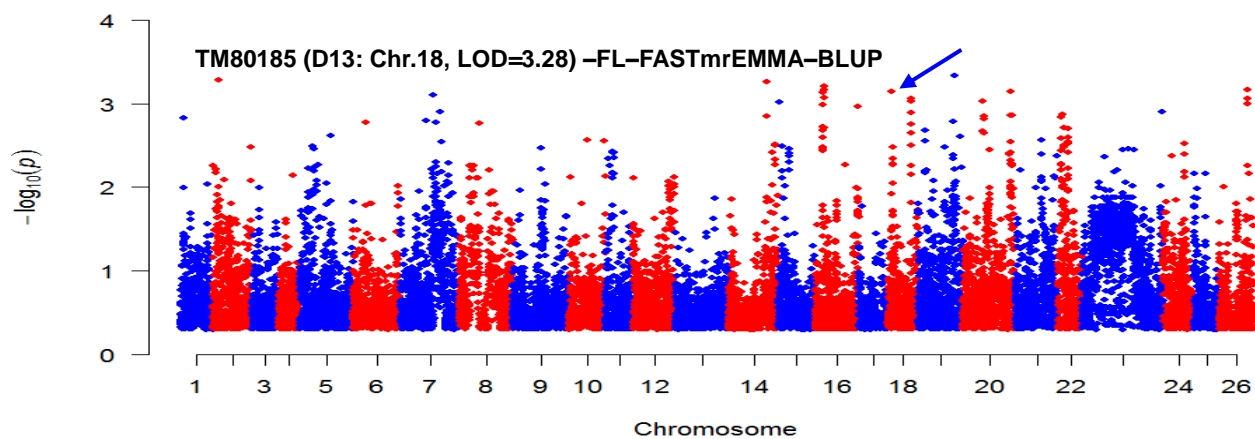

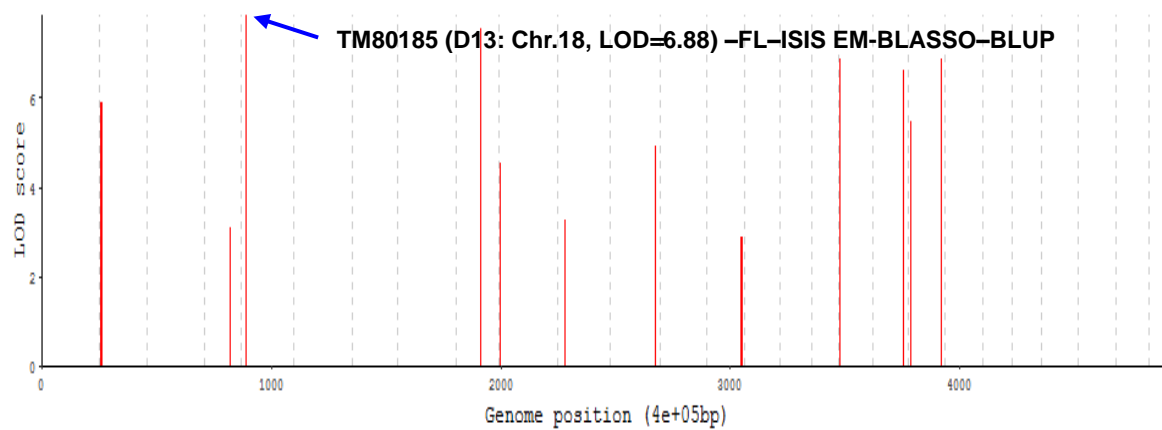

Supplement: Supplementary Figure S2 — QTN, TM80185 (D13), associated with FL, was simultaneously detected in at least two environments, by both single-locus and multi-locus GWASs. [file Data_Sheet_2.PDF]

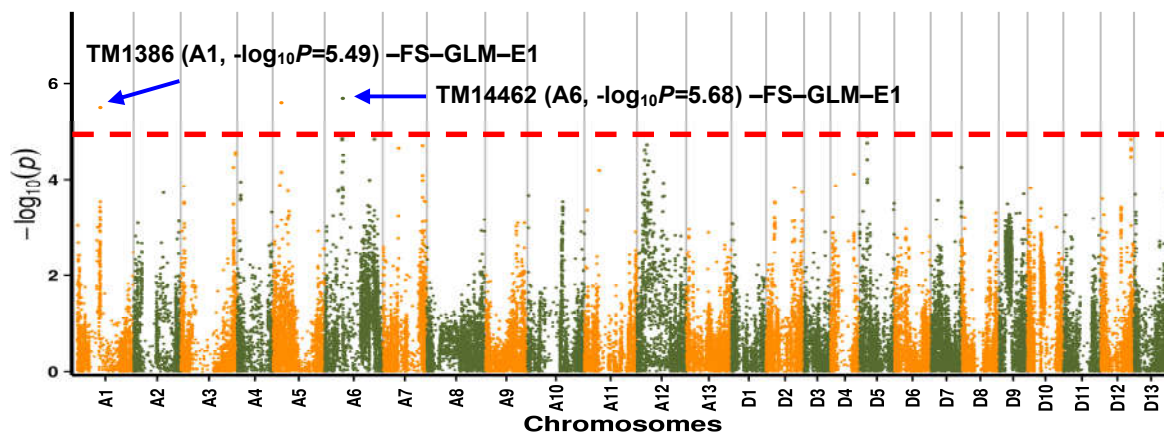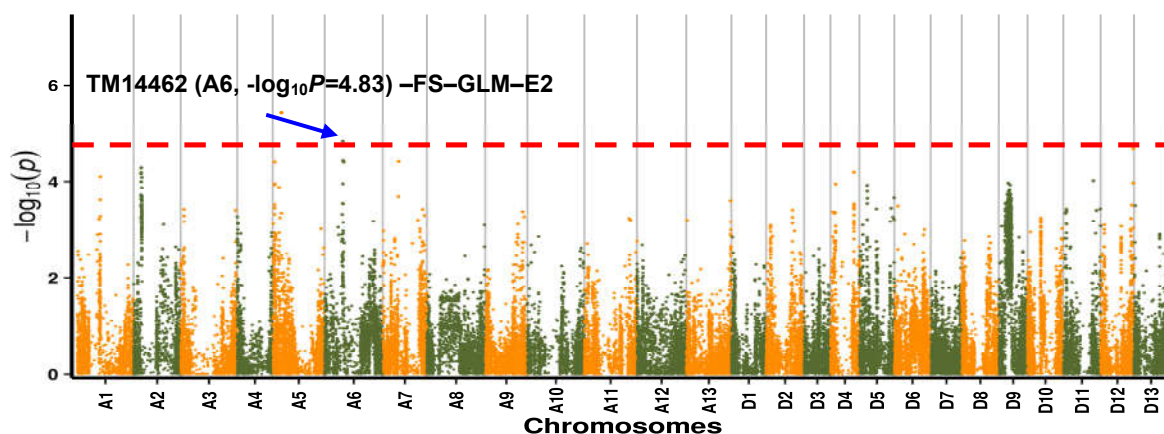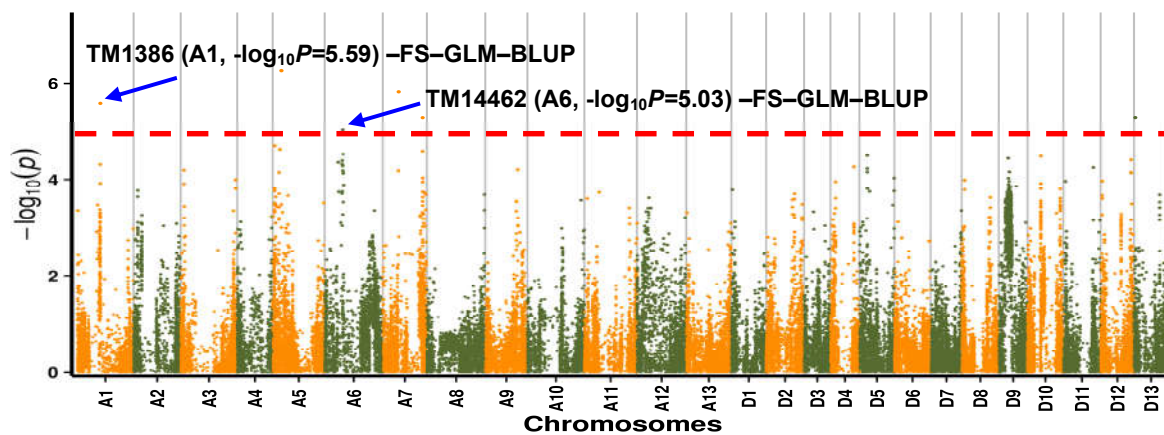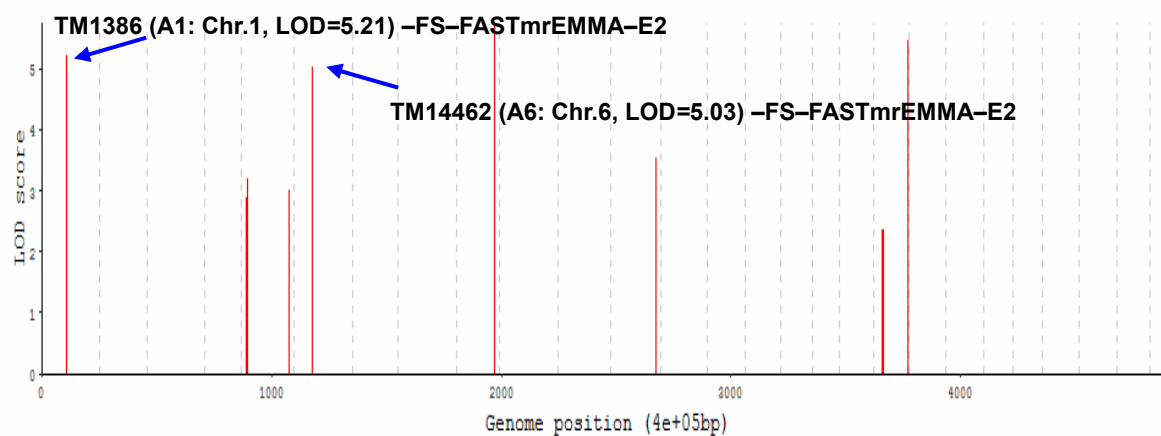

Supplement: Supplementary Figure S3 — QTNs, TM1386 (A1), and TM14462 (A6), associated with FS, were simultaneously detected in at least two environments, by both single-locus and multi-locus GWASs. [file Data_Sheet_3.PDF]

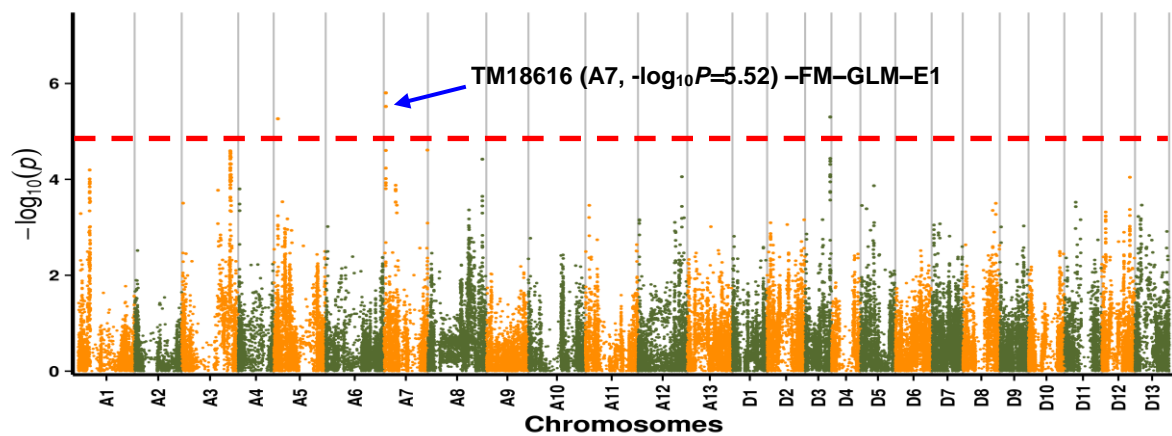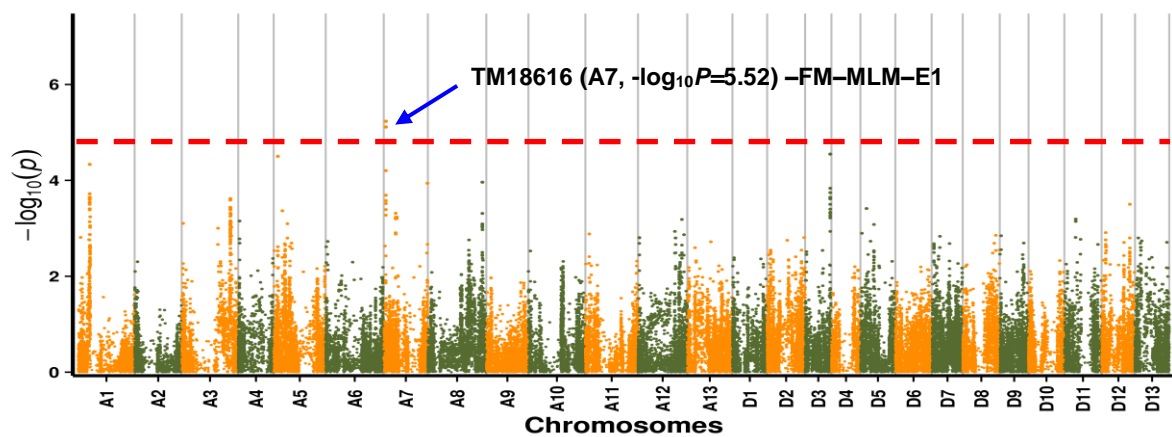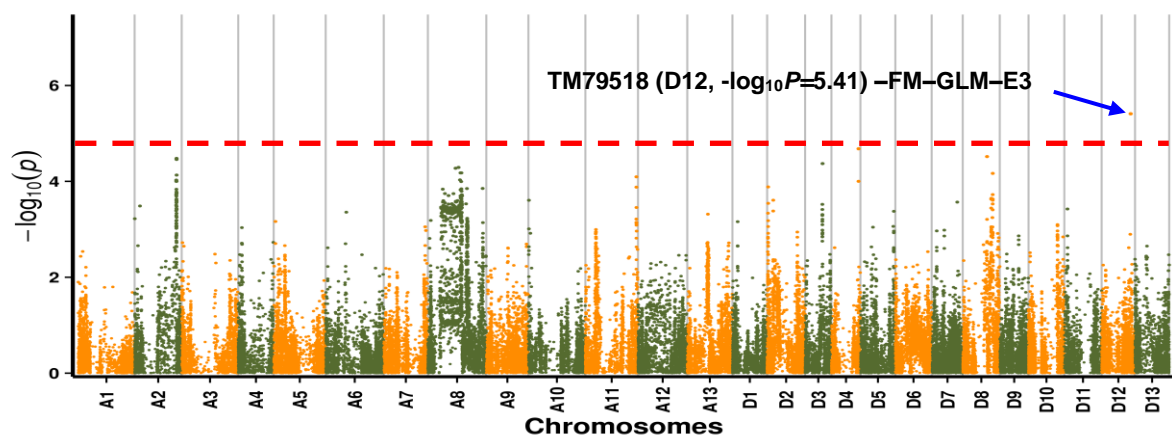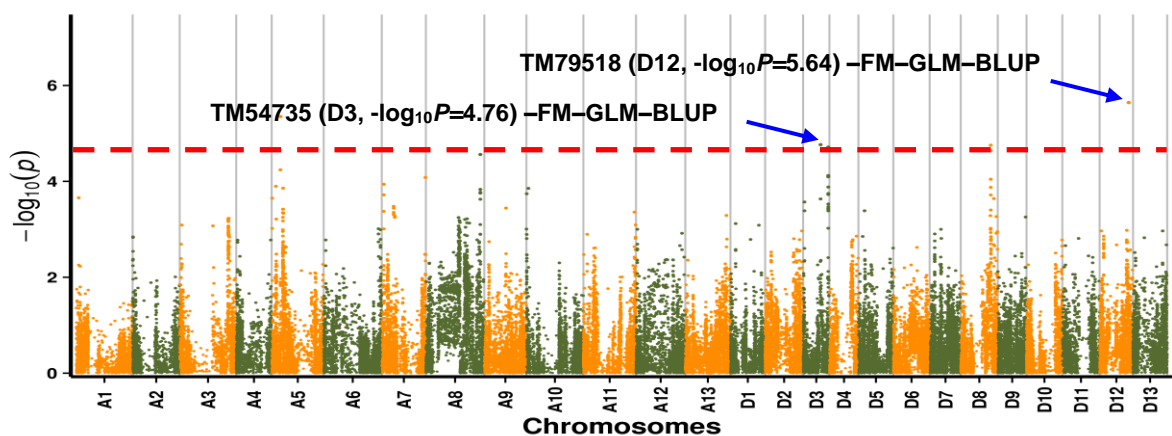

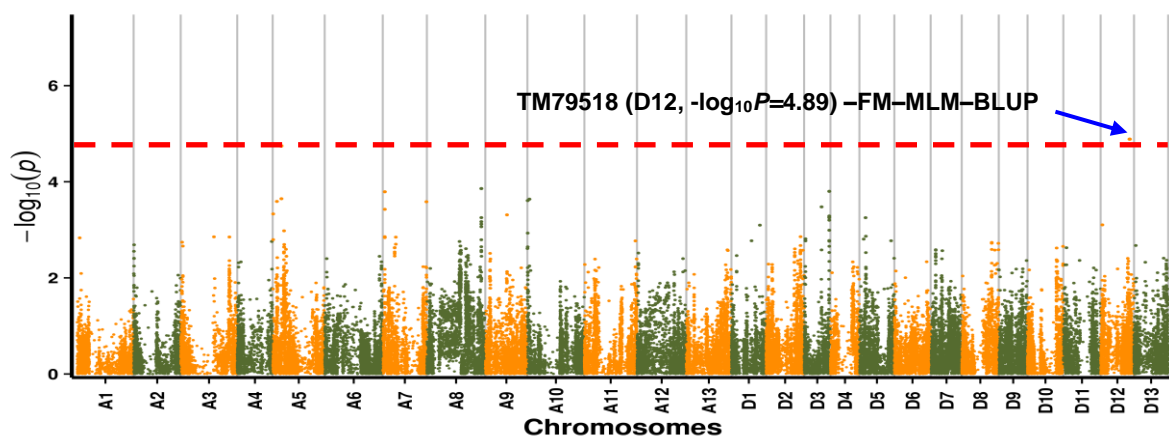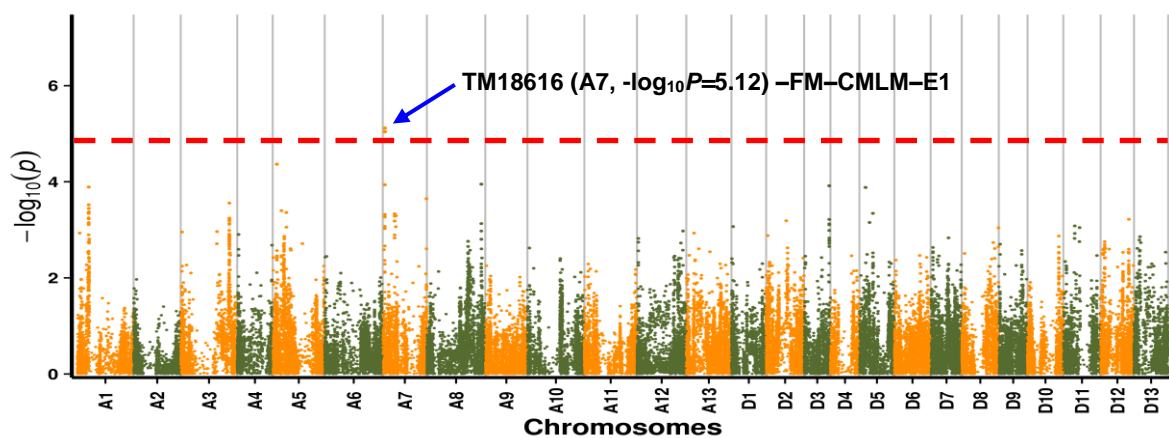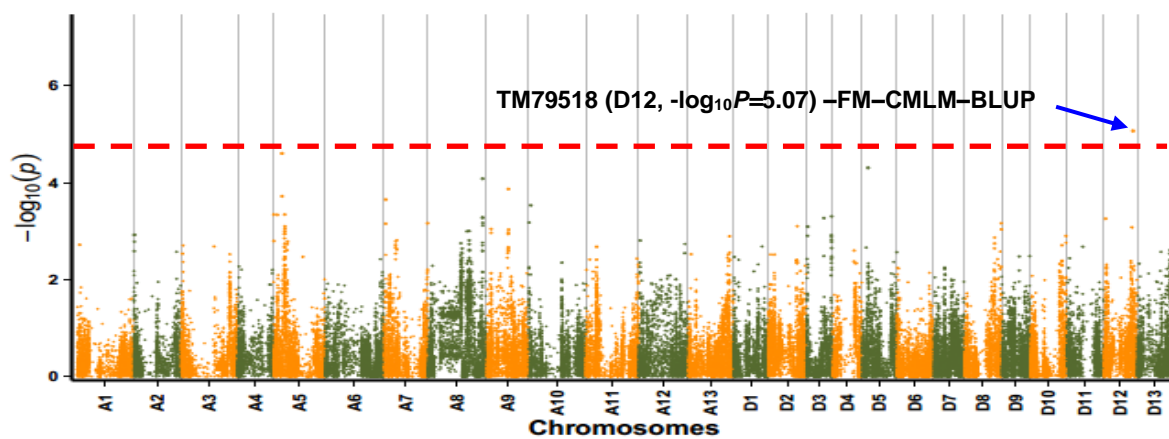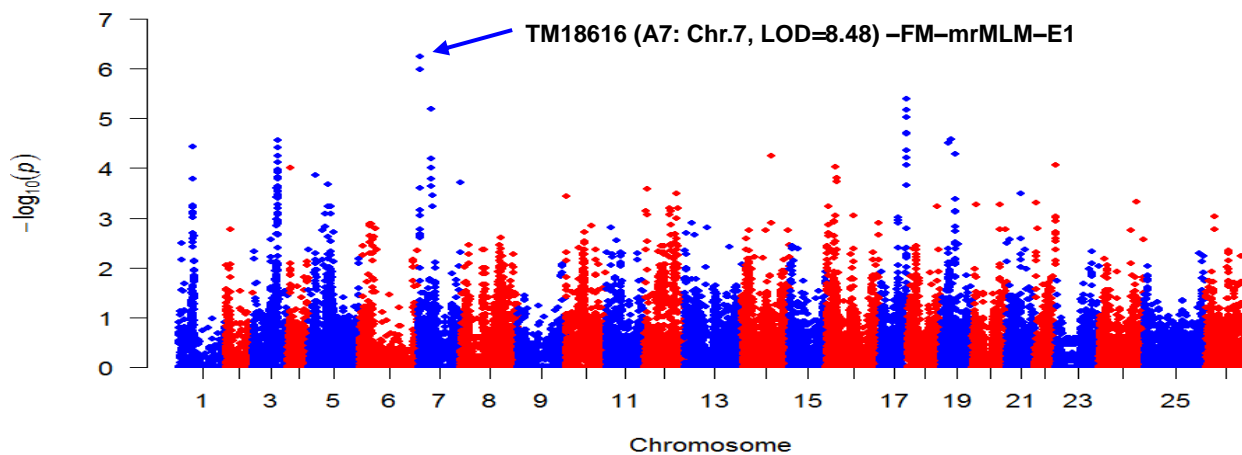

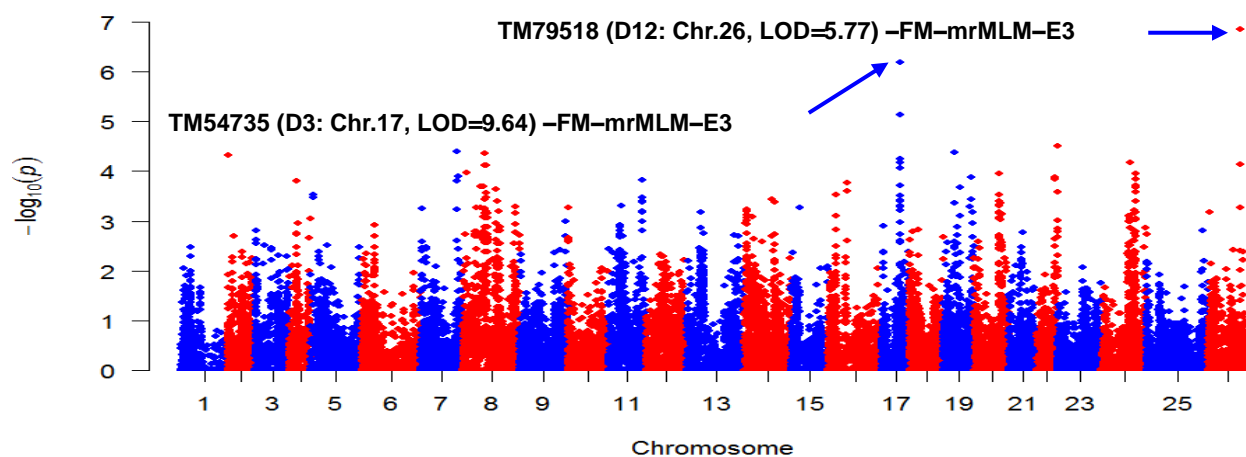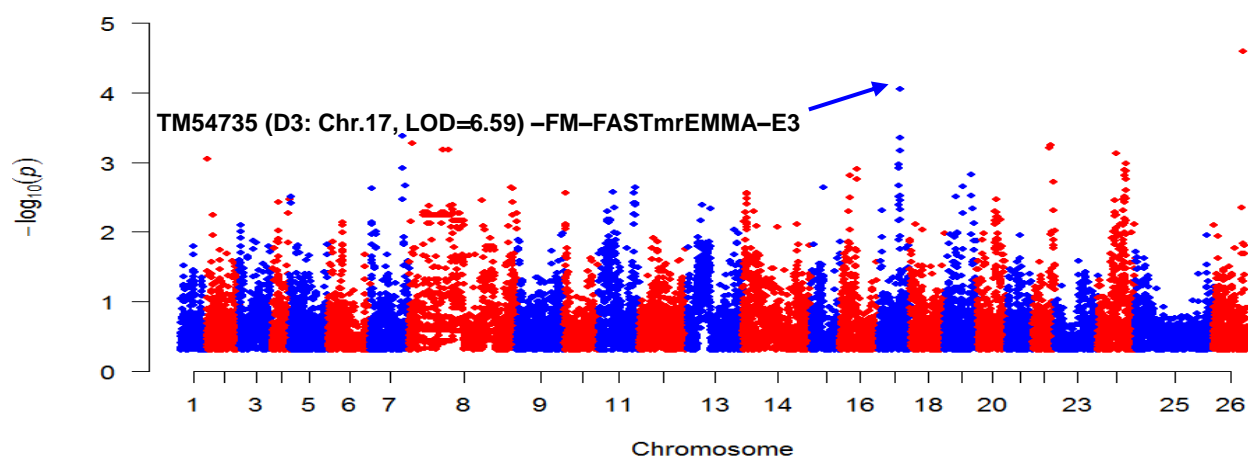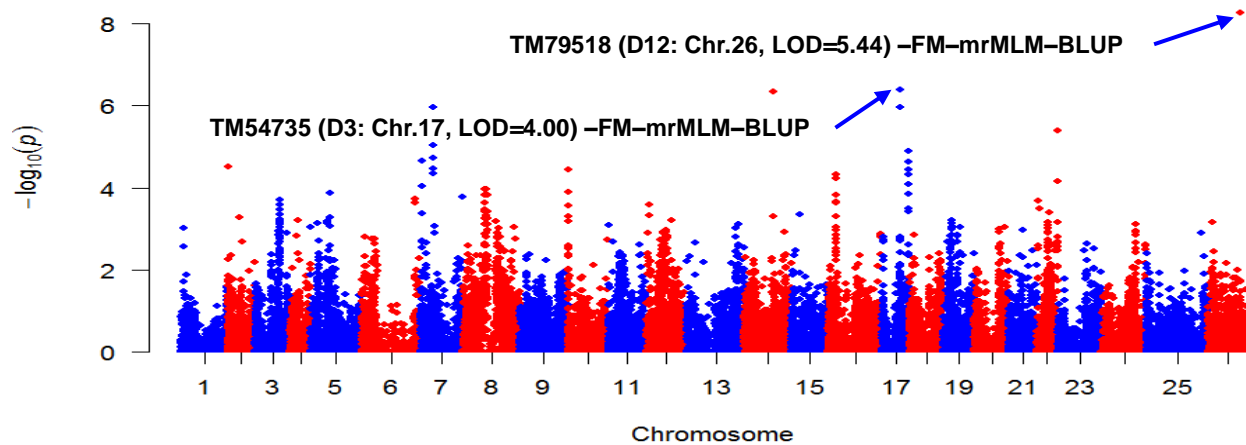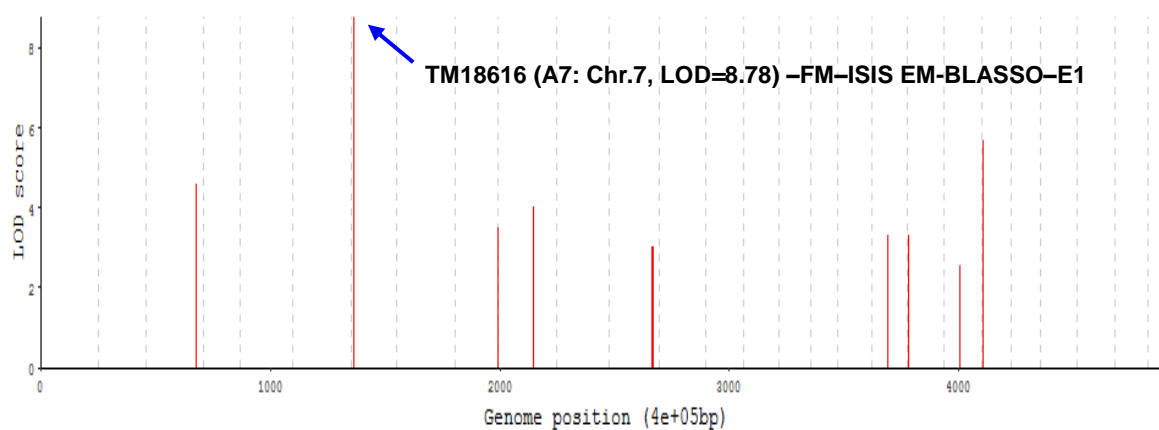

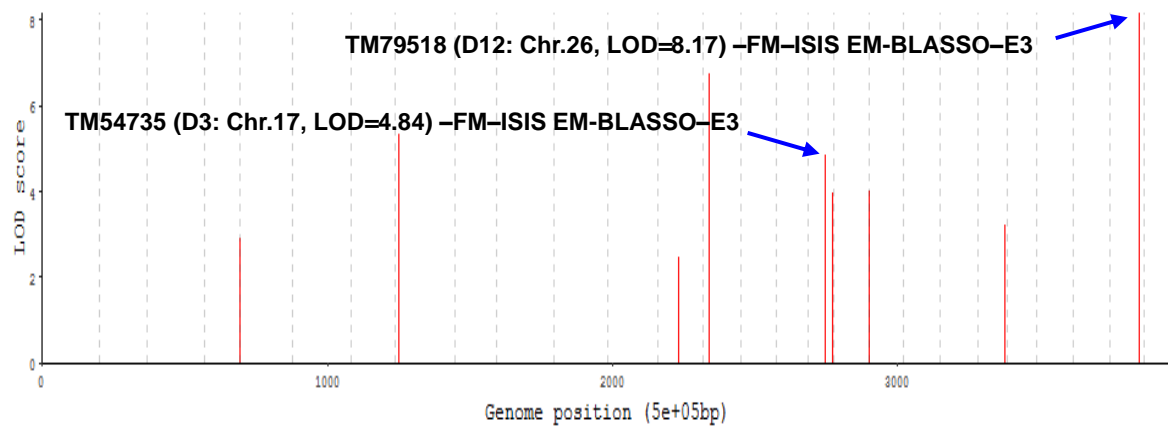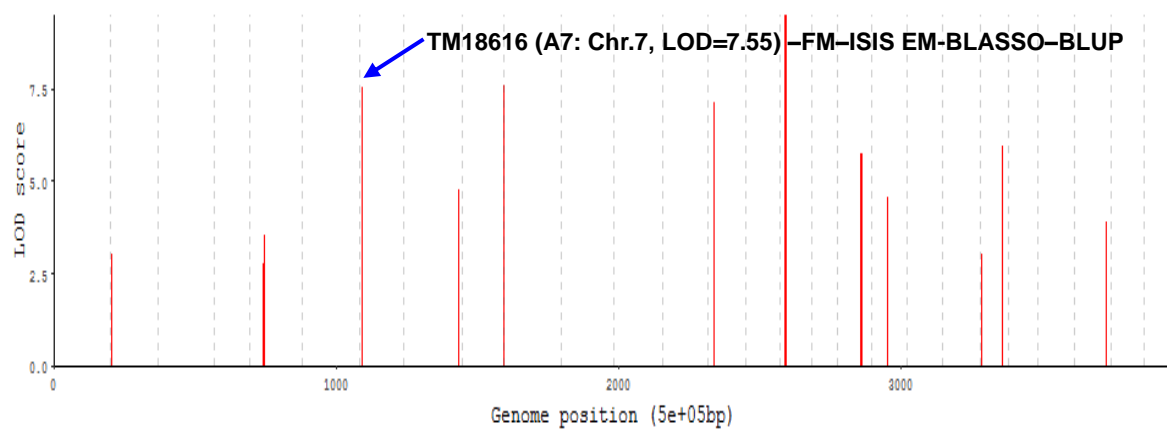

Supplement: Supplementary Figure S4 — QTNs, TM18616 (A7), TM54735 (D3), and TM79518 (D12), associated with FM, were simultaneously detected in at least two environments, by both single-locus and multi-locus GWASs. [file Data_Sheet_4.PDF]

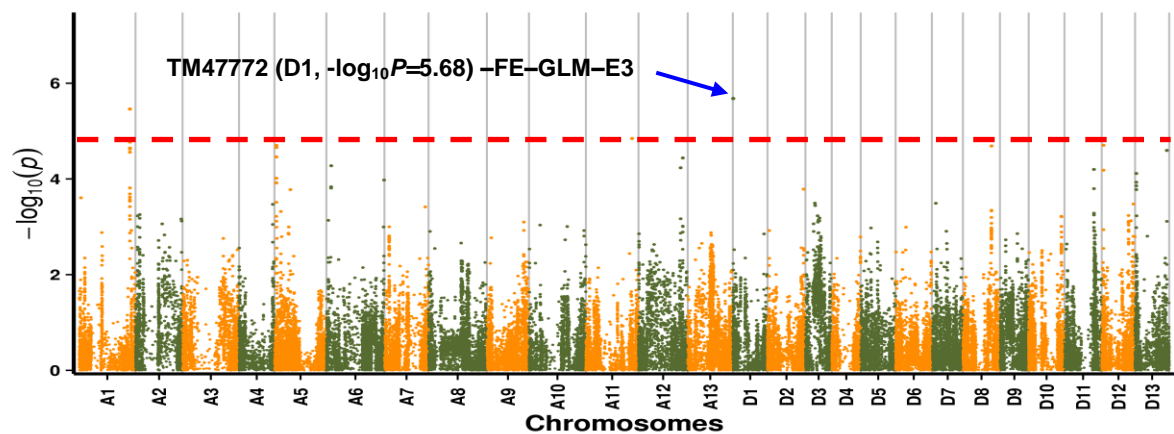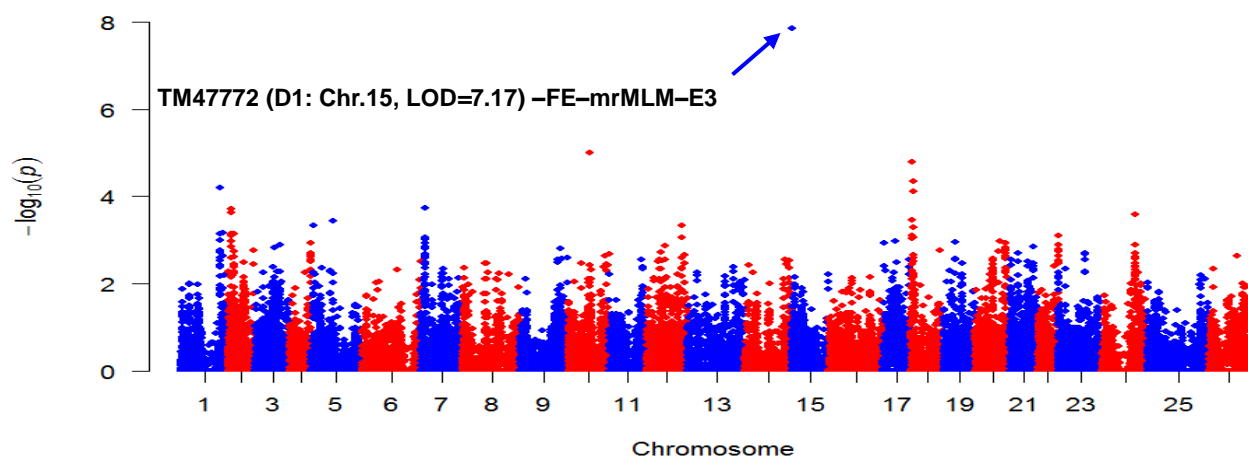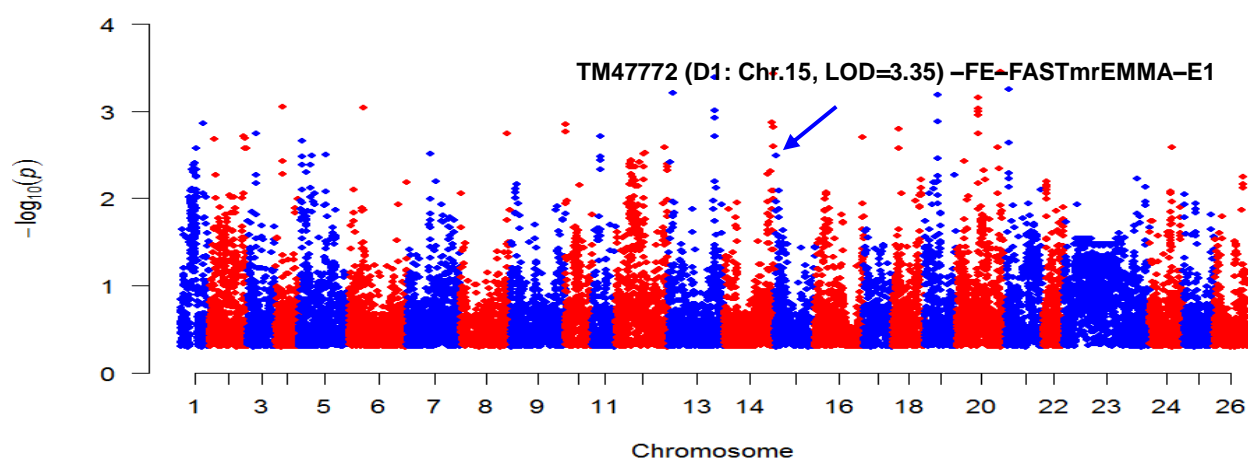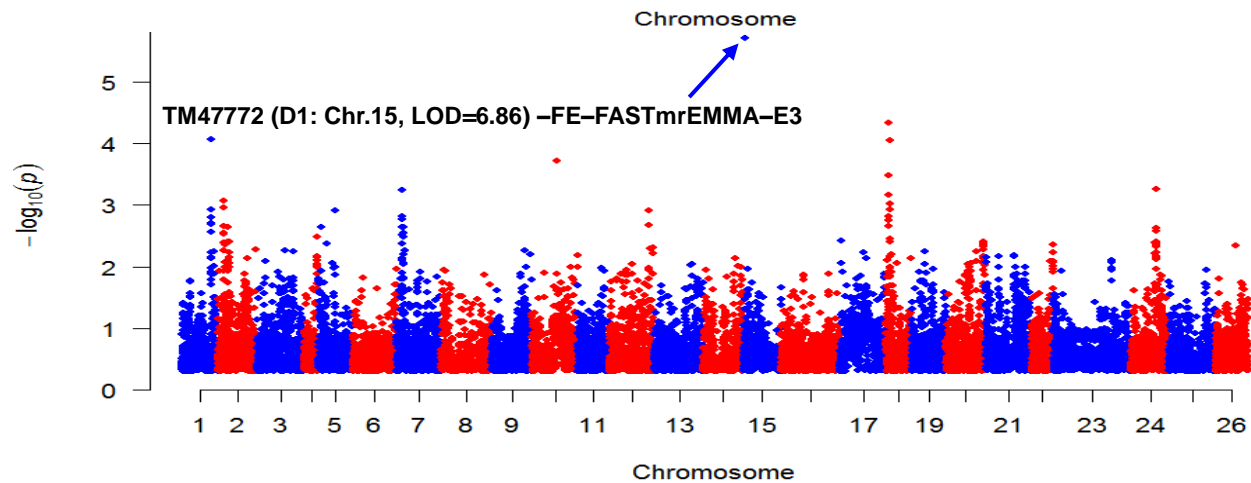

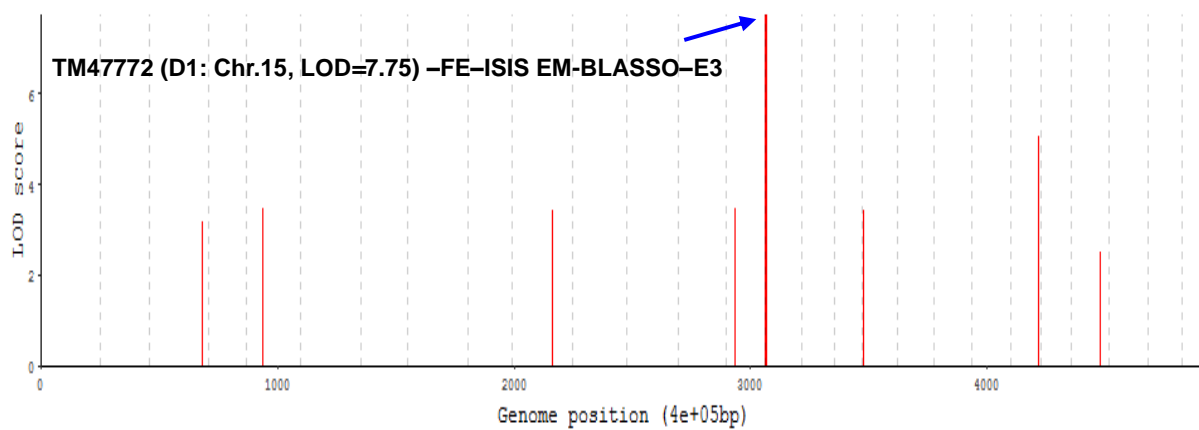

Supplement: Supplementary Figure S5 — QTNs, TM77489 (D12), and TM81448 (D13), associated with FU, were simultaneously detected in at least two environments, by both single-locus and multi-locus GWASs. [file Data_Sheet_5.PDF]

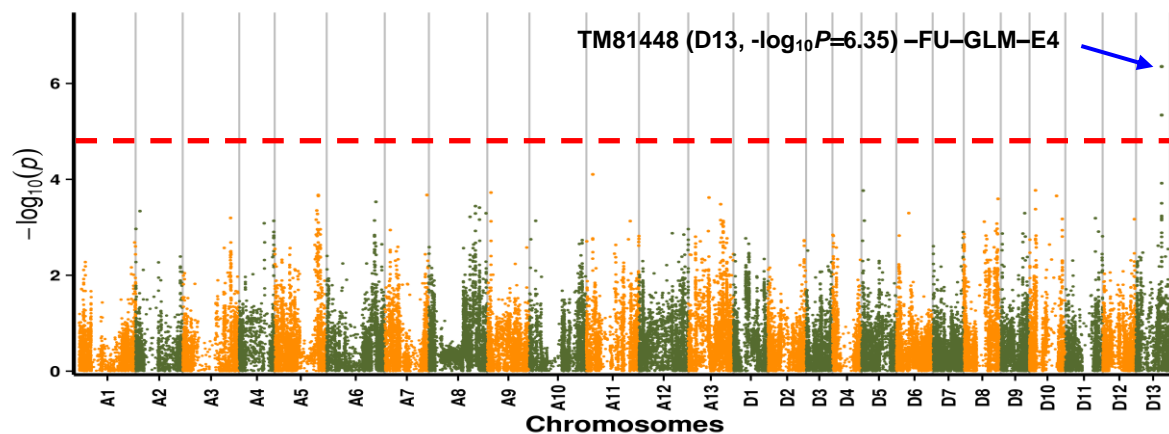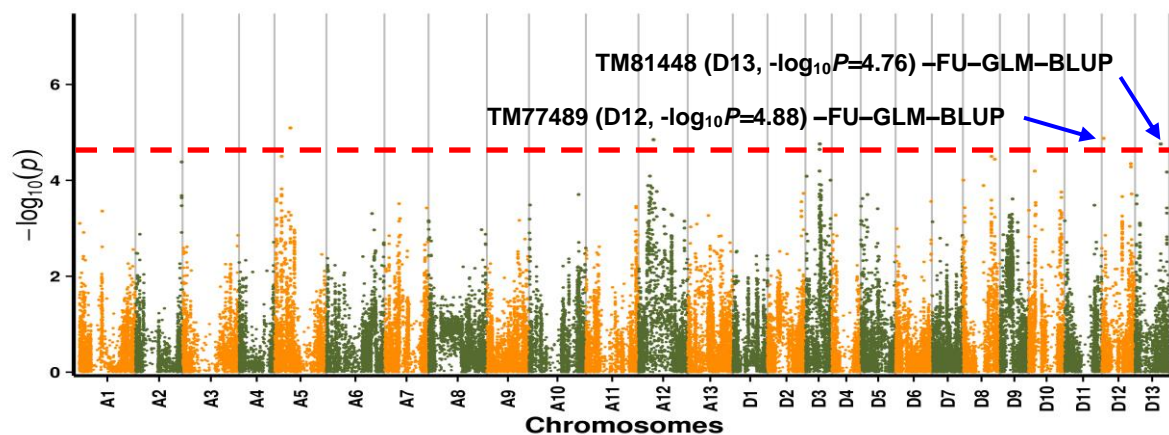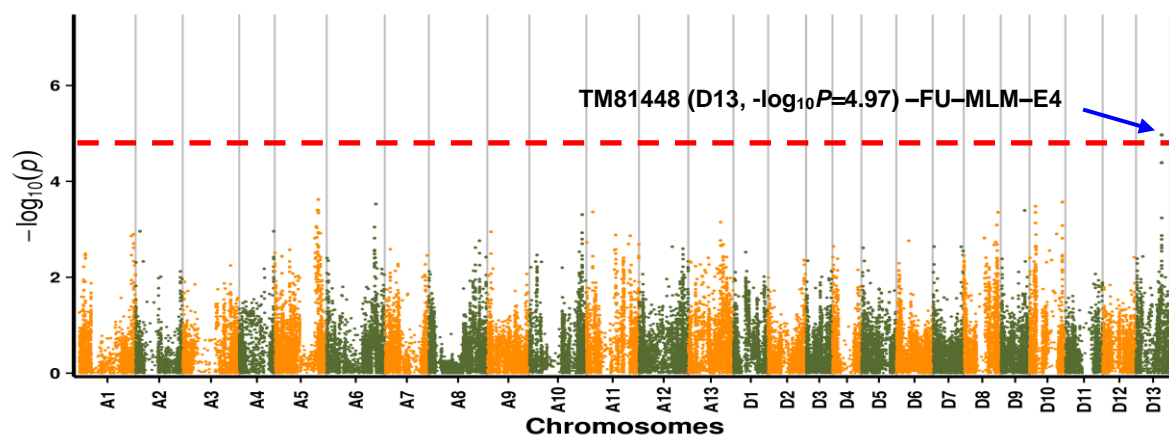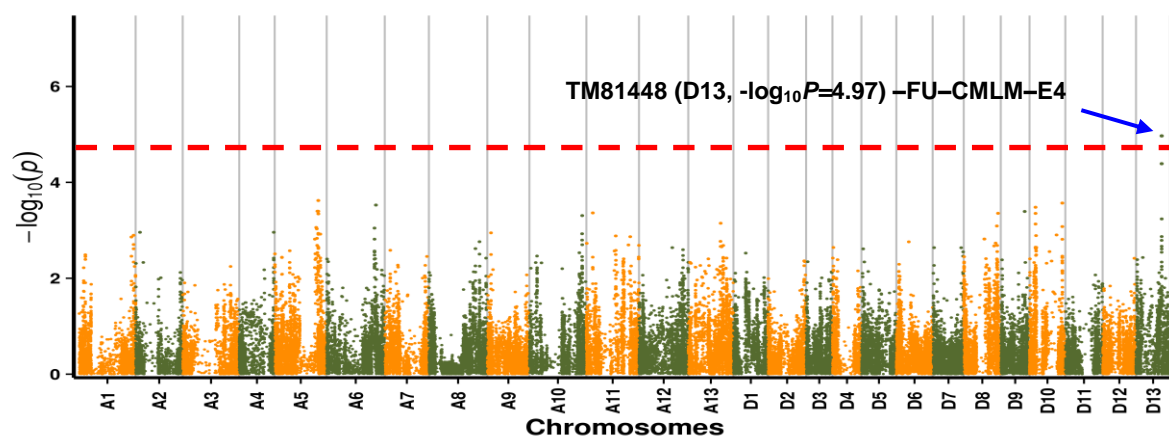

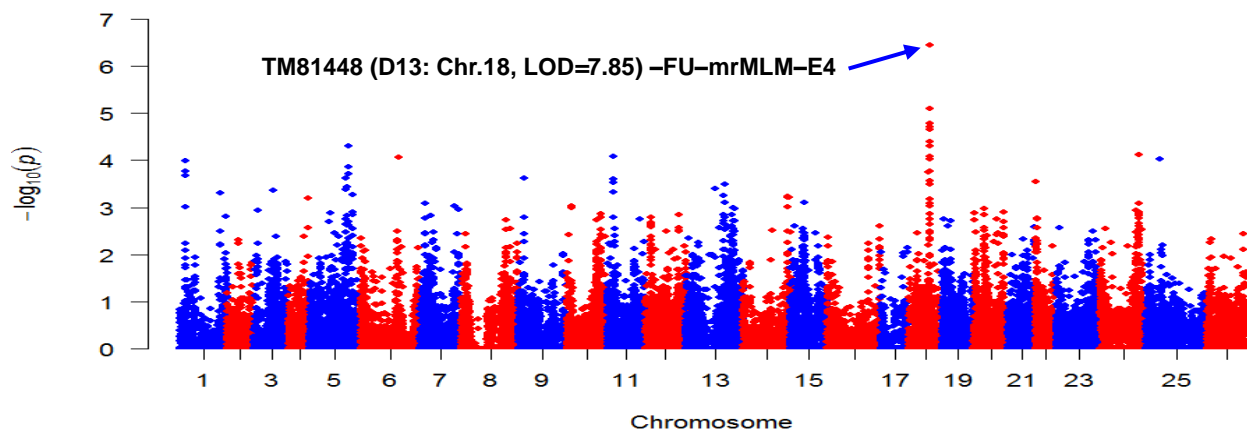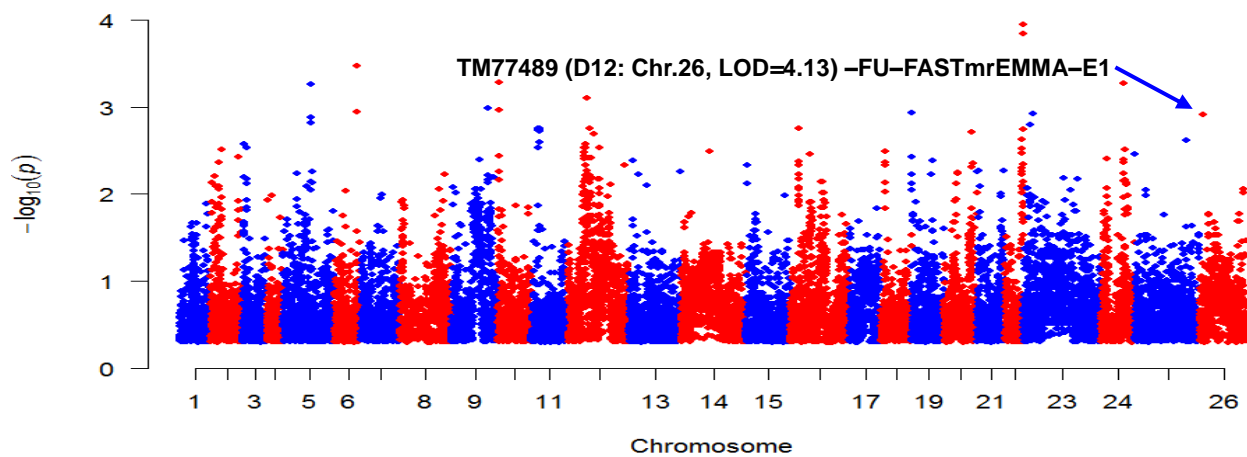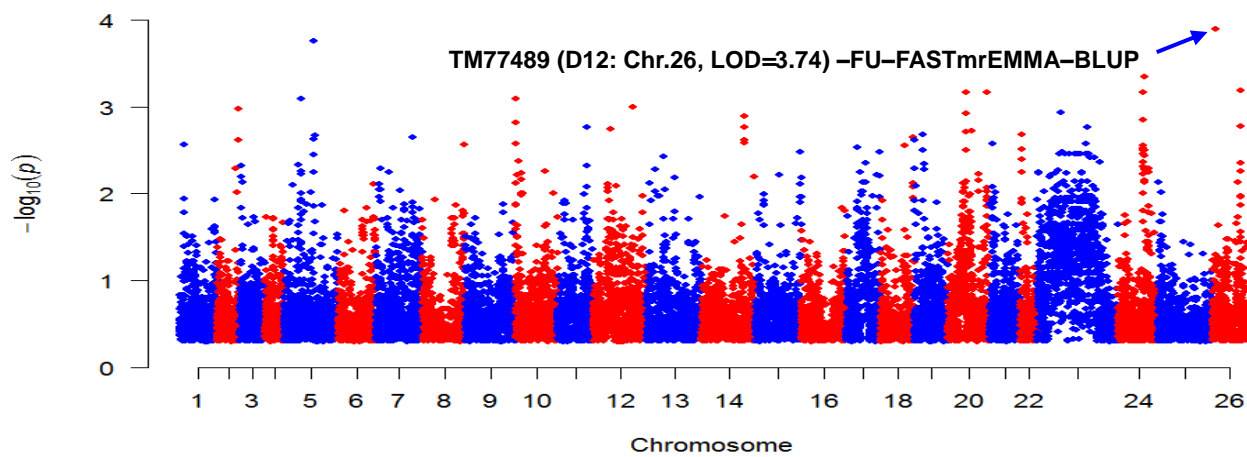

Supplement: Supplementary Figure S6 — QTN, TM47772 (D1), associated with FE, was simultaneously detected in at least two environments, by both single-locus and multi-locus GWASs. [file Data_Sheet_6.PDF]
